# Follicular fluid C3a-peptide promotes oocyte maturation through F-actin aggregation

**DOI:** 10.1186/s12915-023-01760-6

**Published:** 2023-12-08

**Authors:** Ye Yang, Chun Zhao, Beili Chen, Xiaoning Yu, Yuxi Zhou, Danyu Ni, Xiaolan Zhang, Junqiang Zhang, Xiufeng Ling, Zhiguo Zhang, Ran Huo

**Affiliations:** 1 State Key Laboratory of Reproductive Medicine and Offspring Health, Department of Reproduction, Women‘s Hospital of Nanjing Medical University, Nanjing Women and Children‘s Healthcare Hospital, Nanjing, Jiangsu Province 210004 China; 2 State Key Laboratory of Reproductive Medicine and Offspring Health, Department of Histology and Embryology, Nanjing Medical University, Nanjing, China; 3 Reproductive Medicine Center, Department of Obstetrics and Gynecology, the First Affiliated Hospital of Anhui Medical University, Anhui, China; 4 Suzhou Affiliated Hospital of Nanjing Medical University, Suzhou Municipal Hospital, Gusu School,, Nanjing Medical University, Nanjing, China

**Keywords:** C3a-peptide, Human follicular fluid, Liquid chromatography-tandem mass spectrometry, Oocyte in vitro maturation, C3aR, MYO10

## Abstract

**Background:**

Immature cumulus-oocyte complexes are retrieved to obtain mature oocytes by in vitro maturation (IVM), a laboratory tool in reproductive medicine to obtain mature oocytes. Unfortunately, the efficiency of IVM is not satisfactory. To circumvent this problem, we therefore intended to commence with the composition of ovarian follicular fluid (FF), an important microenvironment influencing oocyte growth. It is well known that FF has a critical role in oocyte development and maturation. However, the components in human FF remain largely unknown, particularly with regard to small molecular peptides.

**Results:**

In current study, the follicular fluid derived from human mature and immature follicles were harvested. The peptide profiles of FF were further investigated by using combined ultrafiltration and LC–MS/MS. The differential peptides were preliminary determined by performing differentially expressed analysis. Human and mouse oocyte culture were used to verify the influence of differential peptides on oocyte development. Constructing plasmids, cell transfecting, Co-IP, PLA etc. were used to reveal the detail molecular mechanism. The results from differentially expressed peptide as well as cultured human and mouse oocytes analyses showed that highly conserved C3a-peptide, a cleavage product of complement C3a, definitely affected oocytes development. Intriguingly, C3a-peptide possessed a novel function that promoted F-actin aggregation and spindle migration, raised the percentage of oocytes at the MII stage, without increasing the chromosome aneuploidy ratio, especially in poor-quality oocytes. These effects of C3a-peptide were attenuated by C3aR morpholino inhibition, suggesting that C3a-peptide affected oocytes development by collaborating with its classical receptor, C3aR. Specially, we found that C3aR co-localized to the spindle with β-tubulin to recruit F-actin toward the spindle and subcortical region of the oocytes through specific binding to MYO10, a key regulator for actin organization, spindle morphogenesis and positioning in oocytes.

**Conclusions:**

Our results provide a new perspective for improving IVM culture systems by applying FF components and also provide molecular insights into the physiological function of C3a-peptide, its interaction with C3aR, and their roles in enabling meiotic division of oocytes.

**Supplementary Information:**

The online version contains supplementary material available at 10.1186/s12915-023-01760-6.

## Background

Oocyte maturation is the physiological event that precedes, and is required for, successful fertilization and embryo development. The diplotene-arrested oocyte has an intact nuclear envelope known as the germinal vesicle (GV). Germinal vesicle breakdown (GVBD) and simultaneous extrusion of the first polar body signal the completion of the first meiotic division in human oocytes. Then, the oocyte directly enters meiosis II and is arrested for a second time at metaphase II (MII) before being released at ovulation [1]. In addition to this nuclear maturation process, oocytes also undergo cytoplasmic maturation, including the maturation of various organelles, particularly cortical granules, mitochondria, the endoplasmic reticulum, and cytoskeleton, in preparation for fertilization [2].

Approximately 15–30% of the total number of oocytes retrieved during in vitro fertilization (IVF) treatment are immature. The proportion of immature oocytes even exceeds 50% among patients with advanced age, poor ovarian response, or poly-cystic ovary syndrome [3]. Clinical in vitro maturation (IVM) is a reproductive technology using a suitable environment for enabling oocytes to mature in vitro from ovaries. Clinical IVM has emerged as an attractive treatment due to improving clinical utilization rate of immature oocytes. Despite all this, the maturation rate of clinical IVM is limited, ranging from 51.0% to 57.3%. Gonadotropins, steroids, antioxidants, meiosis inhibitors, and growth factors have been added to IVM medium in attempts to improve the ratio of MII-oocytes [4].

Oocytes develop to maturity within a follicle. Follicles undergo a series of developmental stages, including primordial follicles, primary follicles, secondary follicles (preantral follicles), tertiary follicles (antral follicles), and pre-ovulatory (Graafian) follicles [5]. As follicles progress from secondary follicles to antral follicles, granulosa cells secrete a fluid that accumulates between cells. Large quantities of additional fluid diffuse out of the thecal blood vessels and combine with the secreted fluid to produce what is called follicular fluid (FF). The follicular cavity gradually increases with the development of follicles, and the FF also gradually increases and becomes the microenvironment for oocyte development. Therefore, there are complex paracrine interactions among oocytes, FF, and granulosa cells during follicle growth and development [6].

The FF provides a favorable external environment for the growth and development of oocytes and surrounds the oocyte in an ovarian follicle. It usually contains a large number of hormones, including follicle stimulating hormone (FSH), luteinizing hormone, prolactin, oxytocin and vasopressin, estrogens, and progestogens, and a tremendous amount of other substances, such as growth factors, neuropeptides, and neurotransmitters, providing nourishment for oocyte development. Decreased oocyte quality is often associated with the altered composition of FF [7]. The rate of follicle growth increases dramatically when FF begins to accumulate. Importantly, the oocyte also secretes various products into the follicular microenvironment, which contribute to the maturation of the oocyte itself. Published studies showed that human FF showed diverse metabolic profiles at different follicle developmental stages. Therefore, FF provides valuable information regarding the current growth and development status of follicles. Considering that there is a special intimate proximity between FF and the maturing oocyte and that critical events such as oocyte and follicular maturation, as well as somatic cell-germ cell communication, occur in the FF, it has been accepted that this biological fluid can be used as a unique and non-invasive window to assess oocyte quality [8].

From the clinical perspective, we are looking forward to exploring the altered composition of FF so that oocyte maturation in vitro can be improved. A comprehensive identification of the specific components within FF will help us better understand intrafollicular signal transduction, as well as reveal potential biomarkers of oocyte maturation. However, due to the limitation of the detection approaches used in these studies, some proteins with lower expression levels, especially short peptides, are usually missed.

In this study, we used a strategy combining ultrafiltration and liquid chromatography-mass spectrometry (LC–MS)/MS-based peptidomics to screen and identify novel short peptides responsible for oocyte development. We provided definitive evidence that C3a-peptide, which matches the sequence of anaphylatoxin C3a, a complement component (C3)-derived peptide, significantly improves the rate of both human and mouse oocyte maturation, through direct interaction with receptor C3aR. In mechanism studies, we found that C3aR and β-tubulin co-localized to the spindle and further recruited F-actin to the spindle as well as to the subcortical region of the oocytes by directly binding with Myo10. This series of events promoted the development and maturation of oocytes.

## Results

### Determining the differentially expressed short peptides between human mature and immature follicular fluid by combined ultrafiltration and an LC–MS/MS based peptidomic strategy

**Fig. 1:**
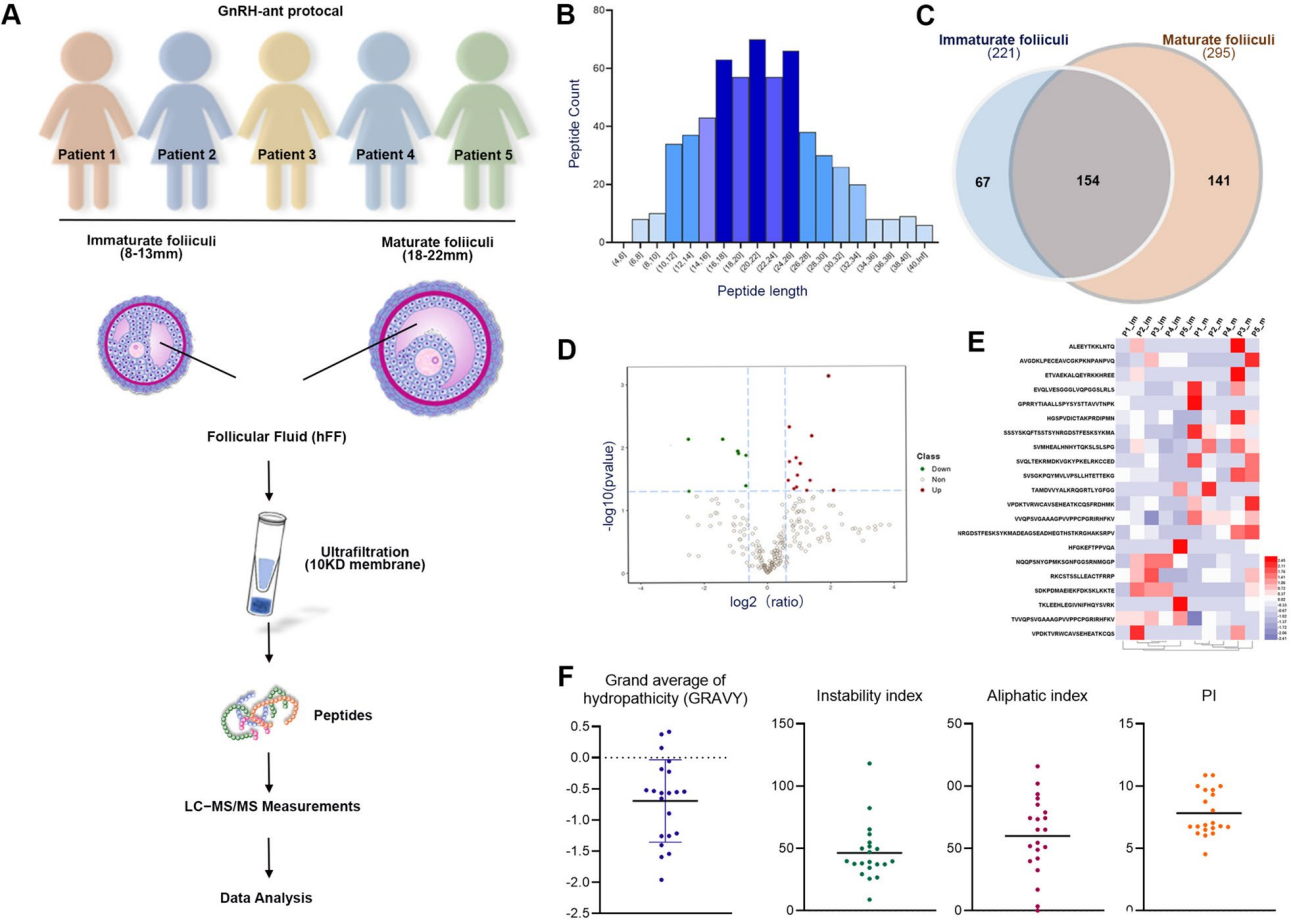
Determining the differentially expressed short peptides between human mature and immature follicular fluid. **A** Workflow of the peptidomic analysis of human follicular fluid (FF). The mature (*n* = 5) and immature (*n* = 5) FF samples were collected according to a GnRH-ant protocol. The collected FF samples were subjected to ultrafiltration to separate the low-molecular-weight fraction (< 10 kDa) containing endogenous peptides (fragments of proteins). The peptides were prepared and analyzed by mass spectrometry. **B** Length distribution of fragments from FF. The x-axis represents the number of amino acids in each peptide, and the y-axis represents the amount of each peptide. **C** Venn plot of the FF peptides identified in this study. The left circle shows peptides from immature FF identified in the experiments (221 in total). The right circle shows peptides from mature FF identified in the experiments (295 in total). A total of 154 differentially expressed peptides between immature and maturated follicles were determined. **D** Volcano map of differentially expressed short peptides. Red represents the upregulated short peptides, and green represents the downregulated short peptides. **E** Heat map comparing hits of 21 candidate short peptides between immature FF 1–5 and mature FF 1–5. The color scale bar is located on the left, and red and blue indicate increased and decreased levels of the identified peptide, respectively. **F** Distributions of differentially expressed short peptides according to their isoelectric point (PI), instability index, aliphatic index, and grand average of hydropathicity (GRAVY)

A mature follicle and an immature follicle were collected from the same women. Five pairs of follicles obtained from five females (10 follicles in total) were subjected to peptide sequence analysis by using LC–MS/MS (Fig. 1A). The lengths of the peptides were in a normal distribution (Fig. 1B). The results from LC–MS/MS (Additional file 1) showed that a total of 362 short peptides were identified, in which 221 peptides were derived from immature FF, and 295 peptides were released by mature follicles. A Venn diagram shows the comparative difference analysis between the two groups, in which the overlapping circle indicates the common short peptides (154), while circles that do not overlap represent characteristic short peptides, with 67 in immature FF and 141 in mature FF (Fig. 1C). By comparing these 154 differentially expressed short peptides with immature FF, we found that 14 short peptides (about 9%) were significantly upregulated in mature FF, corresponding to 14 proteins and three variant sequences, while 7 short peptides (4.5%) were significantly downregulated in mature FF, corresponding to 7 proteins, and 133 short peptides (86%) were unchanged (Fig. 1D). A heat map shows the levels of the 21 differentially expressed short peptides between the five immature FF and five mature FF samples (Fig. 1E). The characteristics of the 21 differentially expressed short peptides, including the grand average of hydropathicity (GRAVY), isoelectric point (PI), instability index, and aliphatic index, are shown in Fig. 1F.

After excluding precursor proteins with unclear functions, four short peptides upregulated in mature FF, namely, apolipoprotein A-I isoform 1 (APPOA1), complement C3 chain A (C3a), immunoglobulin heavy constant gamma 2 (IGHG2), and alpha-2-HS-glycoprotein (FETUA) (aa 20–46), indicated as red characters in Table 1, were selected for subsequent experiments.

**Table 1:** 21 differential short peptides between immature and mature hFF

**Peptide**		**Protein**
**ALEEYTKKLNTQ**	**Up**	**apolipoprotein A-I isoform 1 preproprotein**
**SVQLTEKRMDKVGKYPKELRKCCED**	**Up**	**Chain A, Complement C3**
**SVMHEALHNHYTQKSLSLSPG**	**Up**	**Immunoglobulin heavy constant gamma 2**
**VVQPSVGAAAGPVVPPCPGRIRHFKV**	**Up**	**alpha-2-HS-glycoprotein isoform 1 preproprotein**
ETVAEKALQEYRKKHREE	Up	Apolipoprotein J
EVQLVESGGGLVQPGGSLRLS	Up	Immunoglobulin heavy variable
VPDKTVRWCAVSEHEATKCQSFRDHMK	Up	transferrin
GPRRYTIAALLSPYSYSTTAVVTNPK	Up	Chain A, Transthyretin
HGSPVDICTAKPRDIPMN	Up	serpin peptidase inhibitor clade C member 1
SSSYSKQFTSSTSYNRGDSTFESKSYKMA	Up	fibrinogen alpha chain isoform alpha-E preproprotein
SVSGKPQYMVLVPSLLHTETTEKG	Up	alpha-2-macroglobulin isoform X1
TAMDVVYALKRQGRTLYGFGG	Up	H4
AVGDKLPECEAVCGKPKNPANPVQ	Up	Haptoglobin alpha chain
YNRGDSTFESKSYKMADEAGSEADHEGTHSTKRGHAKSRPV	Up	fibrin alpha C term fragment
NQQPSNYGPMKSGNFGGSRNMGGP	Down	epididymis secretory sperm binding protein
RKCSTSSLLEACTFRRP	Down	transferrin
SDKPDMAEIEKFDKSKLKKTE	Down	TMSB4X protein
TVVQPSVGAAAGPVVPPCPGRIRHFKV	Down	alpha-2-HS-glycoprotein isoform 1 preproprotein
VPDKTVRWCAVSEHEATKCQS	Down	transferrin
TKLEEHLEGIVNIFHQYSVRK	Down	Chain A, S100A12
HFGKEFTPPVQA	Down	hemoglobin beta chain

### The C3a-peptide significantly increases the MII percentage of mouse oocytes without inducing chromosome aneuploidy

**Fig. 2:**
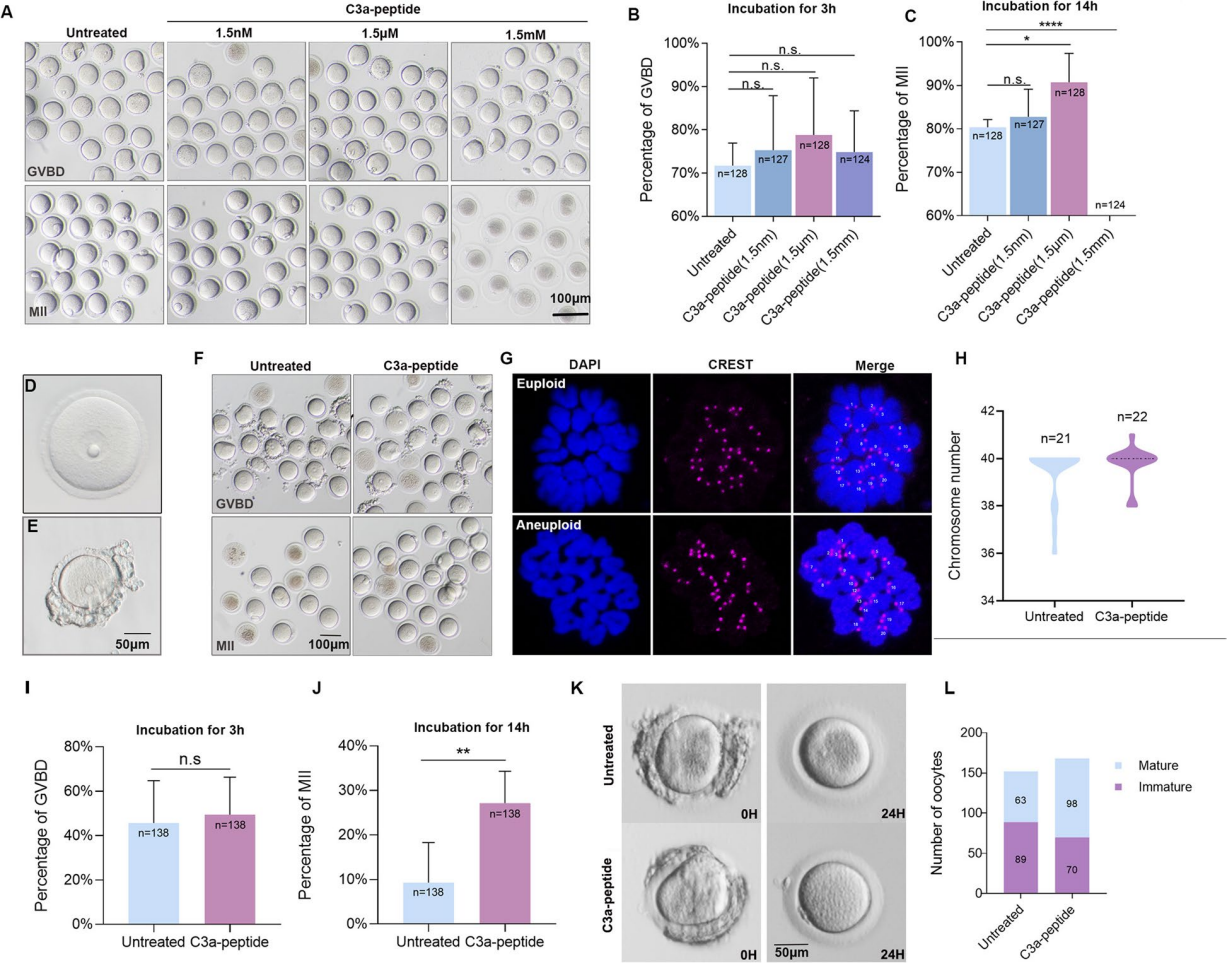
Differentially expressed short peptide, C3a-peptide, promotes oocyte maturation without chromosome aneuploidy change. **A** Morphological change of cultured mouse oocytes in response to treatment with different concentrations of C3a-peptide. **B** Calculation and statistical analysis of the percentage of cultured mouse oocytes at the GVBD stage in response to treatment with different concentrations of C3a-peptide (four independent biological replicates). **C** Calculation and statistical analysis of the percentage of cultured mouse oocytes at the MII stage in response to treatment with different concentrations of C3a-peptide (four independent biological replicates). **D** Mature mouse oocyte. **E** Immature mouse oocyte. **F** Morphological changes of cultured immature mouse oocytes in response to C3a-peptide treatment. **G** C3a-peptide treatment does not induce chromosome aneuploidy of mouse oocytes. Representative images of MII oocyte immunofluorescence stained with DAPI (blue) and anti-CREST (magenta) antibodies showing euploidy and aneuploidy in the oocytes, respectively (left). **H** Calculation and comparison of the chromosome aneuploidy rates between the control and C3a-peptide treatment groups (three independent biological replicates). **I** Calculation and statistical analysis of the percentage of cultured immature mouse oocytes at the GVBD stage in response to C3a-peptide treatment (thirteen independent biological replicates). **J** Calculation and statistical analysis of the percentage of cultured immature mouse oocytes at the MII stage in response to C3a-peptide treatment (thirteen independent biological replicates). **K** Morphological changes of an immature human oocytes after culture for 24 h. **L** Calculation and comparison of human oocyte maturation in response to C3a-peptide treatment (103 patients). Data are presented as the means ± SD. * *P* < 0.05, ** *P* < 0.01, and *** *P* < 0.001 as compared to control cells

To evaluate the effects of these upregulated peptides from mature FF on oocyte development**,** they were synthesized and added to oocyte medium according to their maximum solubility. Subsequently, the growth and development of mouse oocytes were monitored by setting up four experimental groups, untreated, 1.5 nM, 1.5 μM and 1.5 mM treated group. The percentages of oocytes in the GVBD stage and MII stage were calculated (the numbers of oocytes were 128, 127, 128, 124 in four groups respectively). Of the four relatively upregulated peptides, only the C3a-peptide was found to produce an obvious effect on the growth and development of oocytes based on morphological evaluation (Fig. 2A, Additional file 2, Fig. 1S). The optimum concentration of the C3a-peptide was 1.5 μM (Fig. 2A). We found that 1.5 μM of C3a-peptide increased the percentage of oocytes in the MII stage from 80 to 90% (Fig. 2A and C). As for the GVBD stage, albeit not reaching significance, there was an increasing trend with an increasing C3a concentration. Once the concentration of C3a-peptide reached 1.5 mM, the percentage of oocytes at the MII stage was markedly reduced and the cytoplasm became dark, indicating toxic effects (Fig. 2B and C).

However, good quality GV oocytes are not an ideal model for studying the effect of C3a-peptide on the maturation of human oocytes. Our aim was to find a peptide that improved oocytes with poor quality and helped them to mature. Thus, as shown in Fig. 2D and E, the oocytes were divided into two main types: the high quality GV oocytes (clear, moderately granular cytoplasm; small perivitelline space; clear, smooth, colorless zona pellucida) and another oocyte group with a smaller size (rough and surrounded by cumulus cells; an irregular shape typically indicating poor developmental potential). Next, we focused on the effects of C3a-peptide on the oocytes with a smaller size. Although C3a-peptide did not significantly affect the percentage of GVBD oocytes compared with the untreated group (43.4% and 54.6%, *n* = 138) (Fig. 2F and I), the percentage of MII oocytes in the smaller sized group was significantly increased with C3a-peptide treatment, from 9.3% to 27.1% (*n* = 138) (Fig. 2F and J). This suggested that the C3a-peptide significantly increased the percentage of the oocytes at the MII stage as compared with those in the control group. Moreover, to ascertain whether C3a-peptide led to aneuploidy in mouse MII oocytes, chromosome aneuploidy rates were assessed. Specifically, kinetochores were quantified in MII oocytes after immunostaining with mouse anti-centromere CREST antibody (Fig. 2G). This analysis showed that overall, 18% of MII oocytes (4 of 22) were aneuploid in the untreated control group, whereas 19% of MII oocytes (4 of 21) were aneuploid in the C3a-peptide treatment group (Fig. 2H). Altogether, these results indicate that a suitable C3a-peptide concentration could increase the percentage of mouse oocytes at the MII stages, but not at the GVBD stage, and that, more importantly, the C3a-peptide did not elicit statistically significant changes in the chromosome aneuploidy rate during mouse oocyte maturation.

### C3a-peptide supplementation enhances the developmental potential of human immature oocytes without increasing the rate of chromosome aneuploidy

Next, we proceeded to validate the effect of C3a-peptides on human immature oocyte development in a clinical assisted reproductive technology (ART) practice. A total of 320 GV oocytes were collected and were randomly divided into two groups, the control (*n* = 152) and C3a-peptide-treated groups (*n* = 168), for subsequent IVM experiments. After 24 h of culture, we found that the maturation rate of oocytes in the C3a-peptide-treated group was significantly higher than that in the control group based on the observed first polar body (Fig. 2K and L) (58.3% [98/168] vs 41.4% [63/152], *P* = 0.0026)**.** Considering the C3a-peptide as a potential therapeutic drug, a total of 30 human in vitro matured oocytes were subjected to chromosome aneuploidy analysis, including 15 in the control group and 15 in the C3a-peptide-treated group. The results from array comparative genomic hybridization revealed 12 oocytes with a 46, XX karyotype, 1 oocyte with a 47, XX karyotype, and 2 oocytes that could not be successfully karyotyped in the C3a-peptide-treated group, whereas there were 11 oocytes with a 46, XX karyotype, 2 oocytes with a 45, XX karyotype, 1 oocyte with a 43, XX karyotype, and 1 oocyte with a 47, XX karyotype in the control group. Therefore, we did not find any statistical difference in chromosome aneuploidy between the two groups (Table 2). In summary, our data demonstrated that the C3a-peptide indeed increased the percentages of human MII oocytes without increasing the chromosome aneuploidy rate.

**Table 2:** Array CGH of 30 human oocytes in control and C3a-peptide treated groups

Sample		CNV-report
**C3a-peptide**	**1**	46,XX
	**2**	46,XX
	**3**	**46,XX**
	**4**	46,XX, + (6)(q21-q27)(58.70 Mb)(27.068)
	**5**	46,XX
	**6**	46,XX
	**7**	46,XX
	**8**	46,XX
	**9**	46,XX
	**10**	46,XX
	**11**	46,XX,-(6)(q13-q14.1)(5.15 Mb)(12.959)
	**12**	47,XX, + (21)(q11.2-q22.3)(33.59 Mb)(28.978)
	**13**	46,XX
	**14**	NA
	**15**	NA
**Untreated**	**1**	46,XX,-(5)(p15.33-p14.3)(20.68 Mb)(9.965)
	**2**	46,XX
	**3**	46,XX
	**4**	46,XX
	**5**	46,XX
	**6**	46,XX
	**7**	46,XX
	**8**	46,XX
	**9**	46,XX
	**10**	45,XX,-(1)(p36.33-q44)(247.56 Mb)(11.236),-(7)(p22.3-p21.1)(16.78 Mb)(11.498)
	**11**	45,XX,-(16)(p13.3-q24.3)(89.91 Mb)(11.218)
	**12**	46,XX
	**13**	43,XO,-(13)(q11-q34)(95.75 Mb)(10.546),-(16)(p13.3-p11.2)(28.08 Mb)(11.591),-(16)(q11.2-q24.3)(43.59 Mb)(11.006),-(X)(p22.33-q28)(151.12 Mb)(10.858)
	**14**	47,XX, + (3)(p26.3-q29)(197.79 Mb)(32.911)
	**15**	46,XX
	**blank**	NA

### C3a-peptide plays pivotal roles during maturation of oocytes through C3aR

**Fig. 3:**
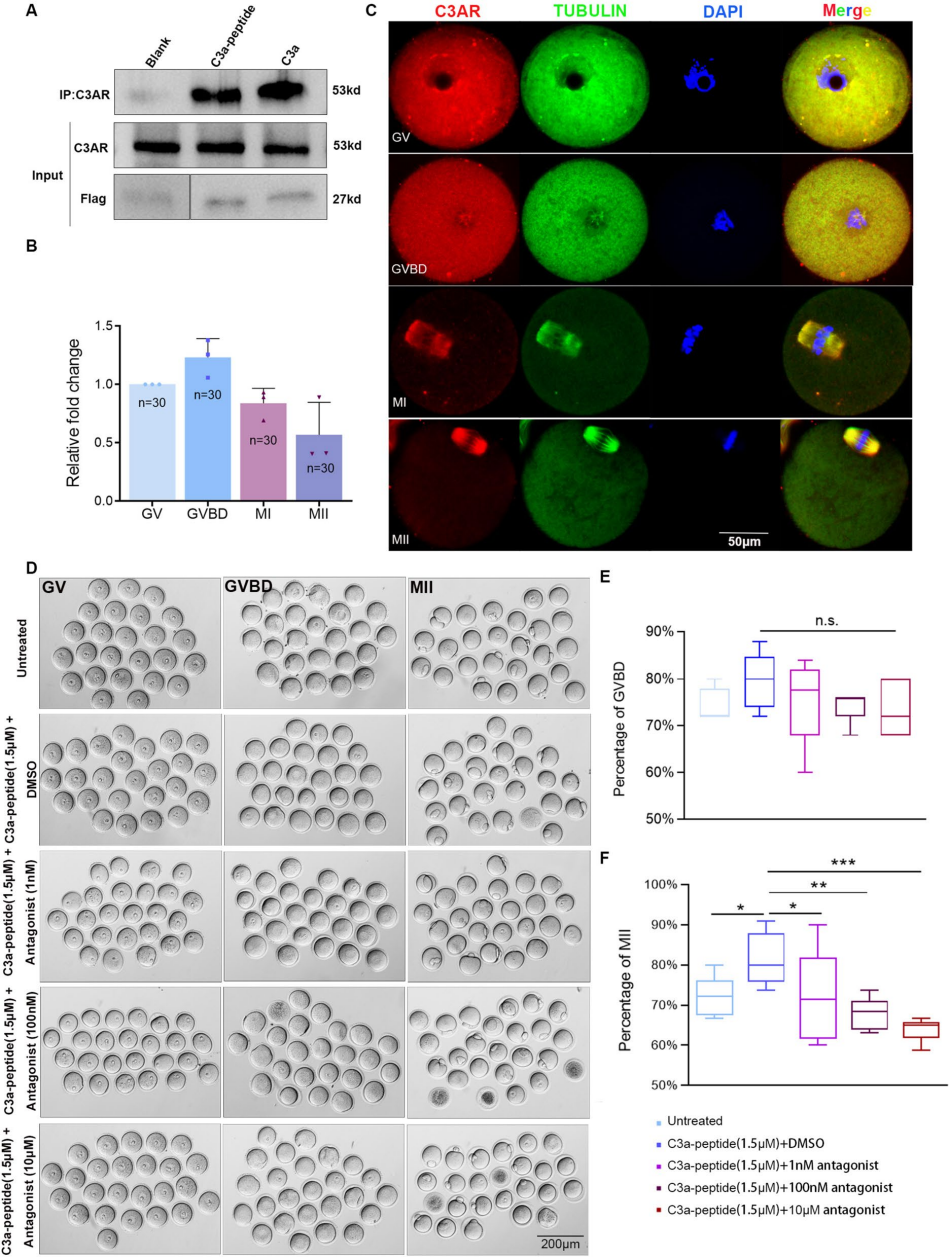
C3a-peptide increases the percentage of oocytes at the MII stage by directly interacting with C3aR. **A** Confirming the direct interaction between C3a-peptide and C3aR by Co-IP. **B** RT-PCR results show the *C3AR1* mRNA expression levels of mouse oocytes at different stages (three independent biological replicates). **C** Double immunofluorescences staining reveals that C3aR and β-tubulin co-localized in the cytoplasm at the GV and GVBD stages and co-localized to spindles at the MI and MII stages (three independent biological replicates). **D** Morphological changes of mouse oocytes during maturation in response to C3a-peptides combined with C3aR antagonist treatment at different concentrations. **E** Calculation and statistical analysis of the percentage of cultured mouse oocytes entering the MII stage in response to C3a-peptide combined with C3aR antagonist treatment at different concentrations (five independent biological replicates). **F** Calculation and statistical analysis of the percentage of cultured mouse oocytes entering the GVBD stage in response to C3a-peptide combined with C3aR antagonist treatment at different concentrations (five independent biological replicates). Data are presented as the means ± SD. * *P* < 0.05, ** *P* < 0.01, and *** *P* < 0.001 as compared to control cells

Each peptide results from the enzymatic processing of large intact protein precursors. We found that the C3a-peptide has high homology between mice and humans, and the sequence of C3a-peptide matched that of the human complement C3a, as determined through a BLAST comparison of the short peptide sequence with the GenBank database (Additional file 3, Fig. S2, A-C). Given that C3a-peptide corresponded to C3a, we wondered whether C3a-peptide exerted the various effects of the native C3a by binding to its receptor, C3aR. A series of experiments were conducted. As shown in Additional file 4, Fig. S3, A, we first observed that after FITC-labeled C3a-peptide was added to the oocyte medium at a concentration of 1.5 μM, positive signals were evenly distributed in the cytoplasm of oocytes, indicating that C3a-peptide penetrated the membrane and successfully entered the cytoplasm of the oocytes. Next, to determine whether the C3a-peptide could bind with C3aR, we constructed the expression plasmids, pcDNA3.1-C3a-peptide-Flag and pcDNA3.1-C3a-Flag. We performed transfection and co-immunoprecipitation (Co-IP) on 293 T cells and used anti-Flag magnetic beads to pull down the expression proteins. As shown in Fig. 3A, C3aR and Flag-tagged proteins were expressed well in the input sample. Moreover, C3aR was present in the immunoprecipitated proteins of 293 T cells transfected with pcDNA3.1-C3a-peptide-Flag and pcDNA3.1-C3a-Flag, indicating that C3a-peptide directly interacted with C3aR. To investigate the expression level and location of C3aR in oocytes, qRT-PCR and immunofluorescence staining were performed. A total of 30 retrieved oocytes were used to extract total RNA in every stage. Compared with the expression level at the GV stage, *C3AR1* (encoding C3aR) mRNA levels reached a peak at the GVBD stage. Then, the transcription of *C3AR1* gradually decreased with maturation and reached the lowest level at the MII stage, suggesting that C3aR was required for mouse oocyte meiotic maturation (Fig. 3B). To clarity the C3aR location, double immunofluorescence staining was conducted. Specially, the specificity of primary antibody C3aR was confirmed through peptide blocking assays (Additional file 4, Fig. S3, D, bottom row). The results from double immunofluorescence staining showed that C3aR positive signals were evenly distributed in the cytoplasm of oocytes at the GV and GVBD stages. Interestingly, after entering the MI and MII stages, C3aR and β-tubulin positive signals were entirely co-localized on the spindles (Fig. 3C). This result was independently verified by using antibodies originating from different companies (Additional file 4, Fig. S3, B). The co-localization phenomenon of C3aR and β-tubulin was also observed in 293 T cells (somatic cell line) (Additional file 4, Fig. S3, C).

To unequivocally confirm that the C3a-peptide played a pivotal role through C3aR, oocytes were treated with both C3a-peptide and the C3aR antagonist SB290157 (0, 1 nM, 100 nM, and 10 μM, 225 oocytes were used in every group). The results showed that C3a-peptide alone increased the percentage of oocytes in the MII stages, and the percentage of MII stages was significantly reduced once combined use of SB290157 with C3a-peptide at any of the concentrations tested. In addition, only 10 μM SB290157 was needed to suppress the otherwise increased GVBD percentage of oocytes induced by C3a-peptide (Fig. 3D–F). Our data suggested that the C3a-peptide played pivotal roles during maturation of oocytes through its receptor C3aR, which might be involved in spindle formation during either meiosis of oocytes or mitosis of somatic cells.

### C3aR morpholino inhibition results in disrupted F-actin aggregation and spindle migration

**Fig. 4:**
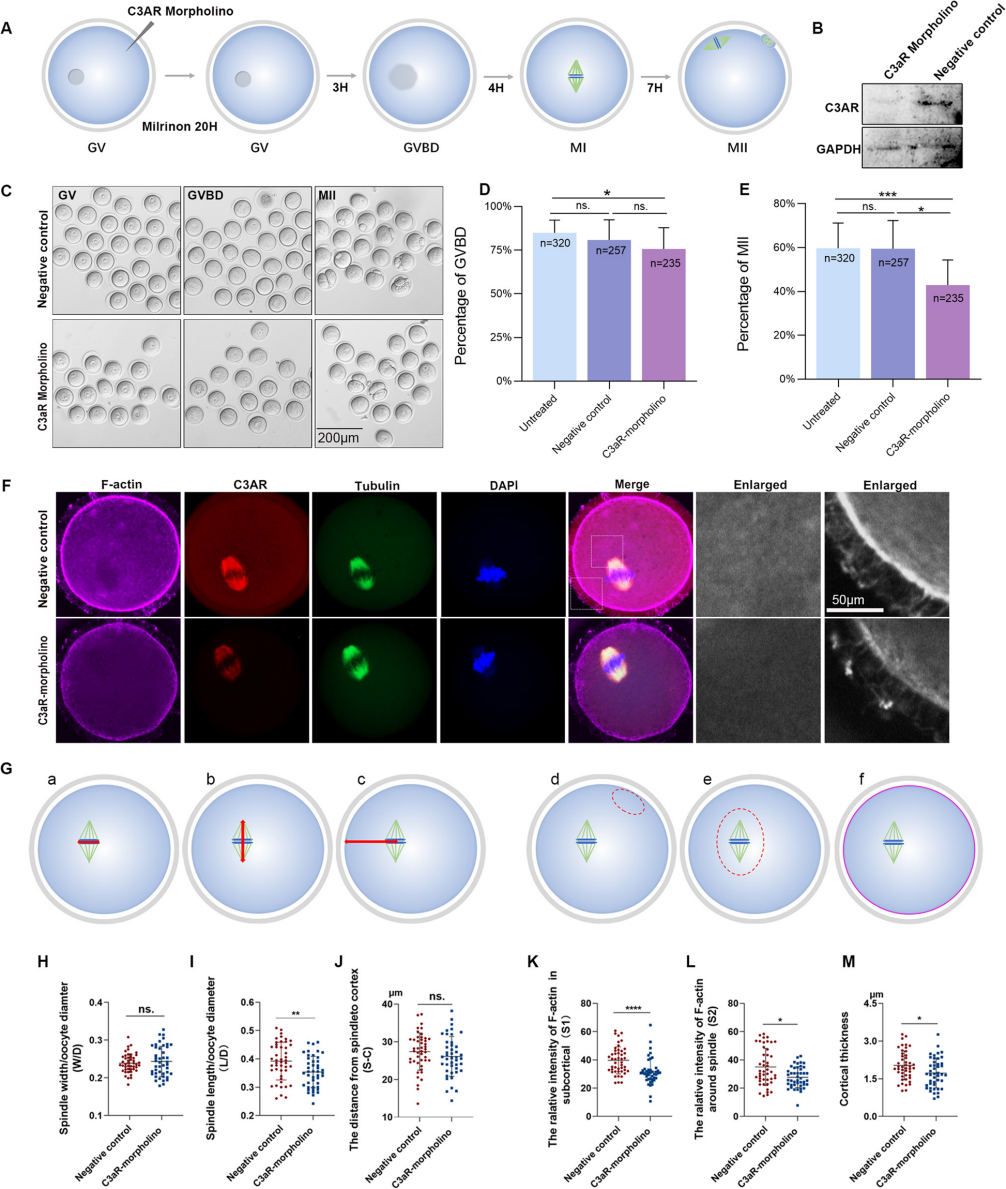
C3aR promotes oocyte maturation by enabling F-actin aggregation and spindle migration. **A** Diagram depicting C3aR morpholino delivery into oocytes. **B** Western blotting shows the validation of the inhibitory efficiency of the C3aR morpholino (two independent biological replicates). **C** Morphological changes of mouse oocytes during maturation in response to C3aR morpholino injection. **D** C3aR morpholinos significantly reduce the percentage of oocytes entering the GVBD stage (five independent biological replicates). **E** C3aR morpholinos significantly reduce the percentage of oocytes entering the MII stage (five independent biological replicates). **F** Triple immunofluorescence staining shows that C3aR morpholino injection suppressed C3aR expression (red) and inhibited F-actin aggregation in the sub-cortical and spindle regions (pink) (three independent biological replicates). **G** Diagram of six quantitative indexes: (**a**) ratio of the spindle width to oocyte diameter (W/D); (**b**) ratio of the spindle inter-polar distance to oocyte diameter (L/D); **(c)** length from the spindle to the cortex (S-C); (**d**) relative intensity of F-actin in the subcortical region (S1); (**e**) relative intensity of F-actin around the spindle (S2); (**f**) cortical thickness. **H** W/D was analyzed and compared between the negative control and C3aR morpholino-injected oocytes. **I** L/D was analyzed and compared between the negative control and C3aR morpholino-injected oocytes. **J** S-C was analyzed and compared between the negative control and C3aR morpholino-injected oocytes. **K** S1 was analyzed and compared between the negative control and C3aR morpholino-injected oocytes. **L** S2 was analyzed and compared between the negative control and C3aR morpholino-injected oocytes. **M** Cortical thickness was analyzed and compared between the negative control and C3aR morpholino-injected oocytes. Data are presented as the means ± SD. * *P* < 0.05, ** *P* < 0.01, and *** *P* < 0.001 as compared to control cells

To further determine the effect of C3aR on mouse oocyte maturation, a C3aR morpholino was introduced. The strategy is shown in the schema graph (Fig. 4A). First, mouse GV oocytes were injected with a C3aR morpholino. The knockdown efficiency was verified by Western blotting (a total of 500 oocytes were retrieved for preparing the protein samples in every group) (Fig. 4B). Then, the growth and development of the oocytes were evaluated, and the percentages at the GVBD and MII stages in untreated (*n* = 320), negative control (*n* = 257) and C3aR- morpholino groups (*n* = 235) were calculated (Fig. 4C). The results showed that although the C3aR morpholino slightly decreased the percentage of oocytes at the GVBD stage compared with the untreated group, there was no statistical difference between the experimental and negative control groups (Fig. 4D). The percentage of oocytes at the MII stage was significantly reduced compared with either the untreated or negative control groups once C3aR was inhibited, while there was no statistical difference between the untreated or negative control groups (Fig. 4E), indicating that C3aR indeed affected mouse oocyte maturation.

Considering that the C3a-peptide might promote mouse oocyte maturation mediated by C3aR by influencing spindle formation or migration, triple immunofluorescence staining with antibodies raised in the same species was used to study the complex distributions of F-actin (pink color), C3aR (red color), and β-tubulin (green color). The results showed that the cloud of F-actin around the spindle disappeared, and the positive signals of a cortex localization of F-actin sharply weakened in C3aR morpholino-injected oocytes compared with the negative control oocytes. Both C3aR and β-tubulin were completely co-located on the spindle. Notably, with C3aR knocked down, the intensity and distribution of β-tubulin did not change (Fig. 4F). To further quantify the influence of C3aR on spindle assembly, migration, and microfilament polymerization, oocytes were divided into two groups (morpholino group, *n* = 44; negative control group, *n* = 46) and six indexes were measured and evaluated, such as the ratio of the short diameter of the spindle (W) to the diameter of the oocyte (W/D) (Fig. 4G-a), the ratio of the long diameter of the spindle (the distance between the two poles, L) to the diameter of the oocyte (L/D) (Fig. 4G-b), the distance from the spindle center to the cortex (S-C) (Fig. 4G-c), the relative F-actin fluorescence intensity in the subcortical region (S1) (Fig. 4G-d), the relative F-actin fluorescence intensity around the spindle (S2) (Fig. 4G-e), and the cortical thickness (red line showed in Fig. 4G-f).

We noticed that there were no statistical differences in the W/D ratio (0.24 ± 0.025 vs 0.24 ± 0.04) (Fig. 4H) and in the mean S-C (27.4 ± 4.96 μm vs 26.09 ± 5.27 μm) (Fig. 4J) between the negative control group and the morpholino group (*P* > 0.05). The mean L/D was slightly shorter (0.35 ± 0.055) in the morpholino group than in the negative control group (0.39 ± 0.067, *P* < 0.01) (Fig. 4I). Moreover, S1 and S2 were significantly weakened with C3aR morpholino treatment. Specifically, S1 in the morpholino group was 31.47 ± 9.322, while the mean S1 in the negative control group was 39.67 ± 9.62 (*P* < 0.001) (Fig. 4K); the mean S2 in the morpholino group was 27.69 ± 7.40, whereas the mean S2 in the negative control group was 34.98 ± 13.14 (*P* < 0.05) (Fig. 4L), suggesting that C3aR inhibition might suppress F-actin polymerization. In addition, the mean cortical thickness in the morpholino group (1.73 ± 0.59 μm) was significantly lower than that in the control group (2.03 ± 0.58 μm) (*P* < 0.05) (Fig. 4M). Altogether, these data indicate that C3aR is partially responsible for polymerization of F-actin and the migration of spindles and that spindle assembly is not affected by C3aR.

### C3a-peptide promotes F-actin aggregation and spindle migration, and this effect of C3a-peptide was attenuated by C3aR morpholino inhibition

**Fig. 5:**
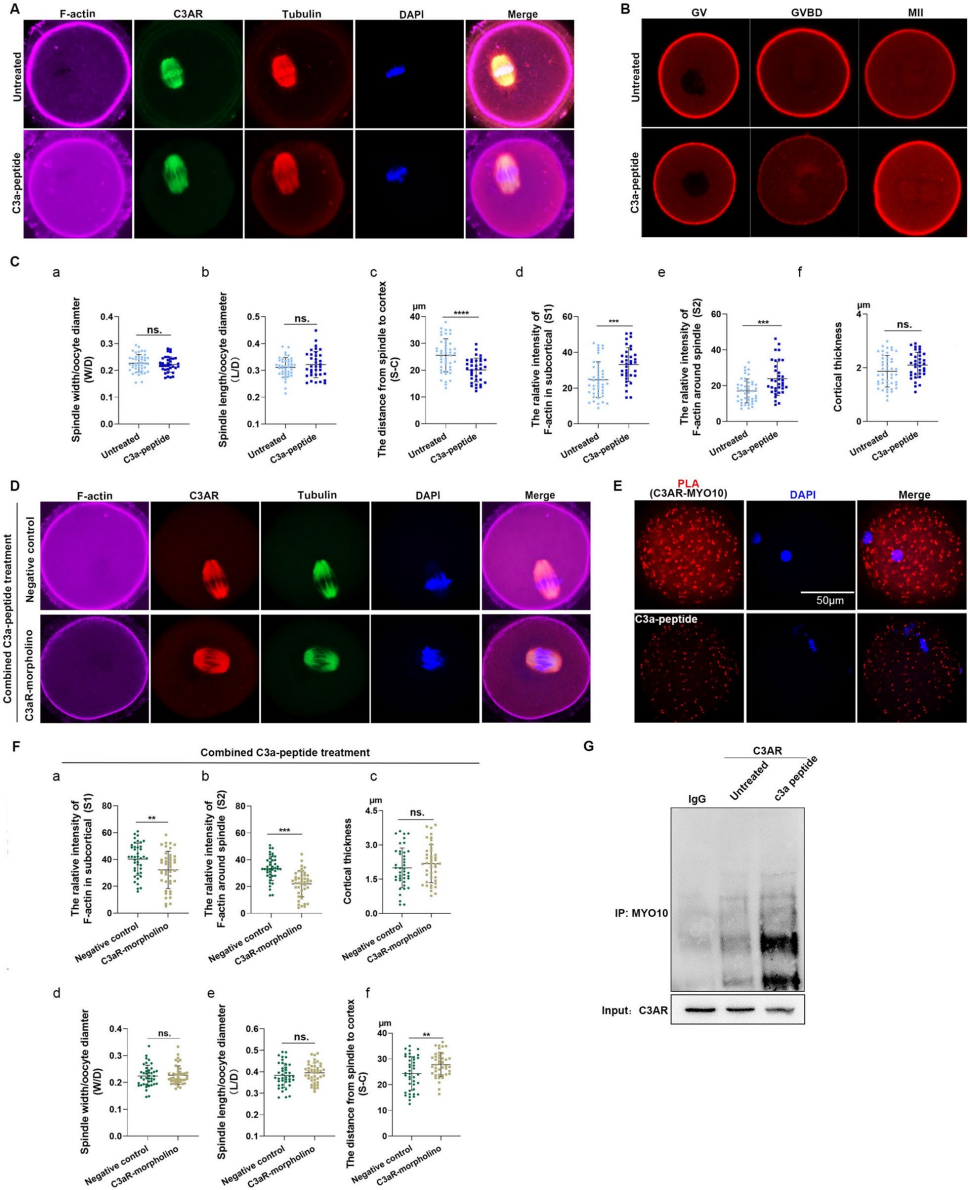
C3a-peptide combined with C3aR promotes F-actin aggregation and spindle dynamics by directly binding to MYO10. **A** Triple immunofluorescence staining shows that C3a-peptide treatment increased C3aR expression (green) and enhanced F-actin aggregation in the cytoplasm, sub-cortical regions, and around the spindle (pink). C3aR and tubulin co-localized to the spindle (three independent biological replicates). **B** F-actin probes in living cells revealed that C3a-peptide treatment enhanced F-actin aggregation in the sub-cortical regions and around the spindle. **C** W/D, L/D, S-C, S1, S2, and cortical thickness were calculated and compared between the untreated and C3a-peptide-treated groups. **D** Triple immunofluorescence staining showed that the enhanced F-actin positive signals in the cytoplasm, sub-cortical regions, and around the spindle (pink) caused by C3a-peptide treatment were restored with a C3aR morpholino injection (three independent biological replicates). **E** Direct interaction between C3aR and MYO10 in mouse oocytes was determined with a proximity ligation assay (PLA) using rabbit anti-C3aR and anti-MYO10 antibodies. After staining, the oocytes were imaged by confocal and differential interference contrast (DIC) microscopy. Scale bar, 20 μm. **F** W/D, L/D, S-C, S1, S2, and cortical thickness were calculated and compared between the C3a-peptide-treated group and the combined C3a-peptide-treated and C3aR morpholino-injected group. **G** Confirming the direct interaction between C3aR and MYO10 by Co-IP (three independent biological replicates). Data are presented as the means ± SD. * *P* < 0.05, ** *P* < 0.01, and *** *P* < 0.001 as compared to control cells

To reveal the changes of F-actin polymerization and distribution during the development of mouse oocytes cultured with C3a-peptide, we first constructed F-actin probes in living cells, pcDNA3.1-3xmscarleti_Utrch, which could specifically bind to F-actin without changing its kinetic characteristics. The results showed that at the GVBD stage, F-actin polymerization around the spindle was more obvious in C3a-peptide-treated oocytes than in untreated oocytes and that at the MII phase, the fluorescence intensities of microfilaments in the cortical region and cytoplasm, especially surrounding the spindle, were stronger in the C3a-peptide-treated oocytes than in the untreated group (Fig. 5B). These results suggest that C3a-peptide promoted F-actin polymerization and regulated the distribution of F-actin. With delivery of C3a-peptide, triple immunofluorescence staining showed enhanced F-actin positive signals in the sub-cortical region and around the spindle compared with the untreated group (Fig. 5A).

Unlike the C3aR morpholino injection group, the fluorescence intensities of subcortical F-actin and around the spindle were stronger in the C3a-peptide group than in the untreated group (*P* < 0.001). The fluorescence intensity S1 in the C3a-peptide group was 33.16 ± 9.45, and in the untreated group, it was 24.69 ± 10.00 (Fig. 5C-d). S2 in the C3a-peptide group was 23.92 ± 9.50, and in the untreated group, it was 17.04 ± 6.70 (Fig. 5C-e). Furthermore, compared with the untreated group (n = 45), a much shorter S-C was found in the C3a-peptide group (25.54 ± 6.20 μm vs 20.07 ± 4.4 μm, *P* < 0.001, respectively) (*n* = 35) (Fig. 5C-c). There were no significant changes in the W/D (0.23 ± 0.03 vs 0.22 ± 0.03), L/D (0.31 ± 0.04 vs 0.32 ± 0.05), or cortical thickness (1.87 ± 0.59 vs 2.10 ± 0.48) between the untreated group and C3a-peptide group respectively (Fig. 5C-a, b, and f).

Importantly, as expected, the enhanced relative fluorescence intensities of F-actin in the sub-cortical region and surrounding the spindle, as well as the upregulated expression of C3aR caused by C3a-peptide, significantly decreased with C3aR morpholino administration (Fig. 5D). Quantitatively, the altered S1, S2, and S-C intensities caused by the C3a-peptide were partially restored by injecting the oocyte with C3aR morpholino; the mean S1 declined from 40.18 ± 12.0 to 32.2 ± 13.87 (*P* < 0.01) (Fig. 5F-a); the mean S2 decreased from 33.5 ± 9.00 to 22.0 ± 9.65 (*P* < 0.001) (Fig. 5F-b); and the S-C was lengthened from 24.3 ± 6.52 μm to 27.8 ± 4.65 μm (*P* < 0.01) (Fig. 5F-c) between the negative control and morpholino-treated groups. There were still no obvious changes in the W/D (0.22 ± 0.040 vs 0.23 ± 0.035, respectively, *P* > 0.05) (Fig. 5F-d), L/D (0.38 ± 0.056 vs 0.39 ± 0.045, respectively, *P* > 0.05) (Fig. 5F-e), and the cortical thickness (1.975 ± 0.87 vs 2.178 ± 0.83, respectively, *P* > 0.05) (Fig. 5F-f) between the negative control group (*n* = 41) and the C3aR morpholino-treated group (*n* = 43). To summarize, these observations indicated that the C3a-peptide recruited F-actin to the subcortex and around the spindle, accelerating F-actin aggregation and spindle migration, but did not affect spindle formation and that C3a-peptide exerted its biologic role primarily through its receptor, C3aR.

### C3a-peptide/C3aR promotes F-actin aggregation and spindle migration during oocyte maturation by interacting with Myo10 but does not impact spindle formation

Next, we explored how C3a-peptide/C3aR influenced spindle migration and F-actin aggregation. Published studies showed that MYO10 directly links F-actin to spindle microtubules in mouse oocytes and thus participates in spindle off-centering. Proximity ligation assays (PLAs) provide highly specific and sensitive in situ detection of protein interactions within a complex [9]. We therefore investigated whether there was a direct interaction between MYO10 and C3aR by conducting PLAs and Co-IP assays. The results showed that the intensities of positive signals in C3a-peptide-treated oocytes were stronger than those in normal oocytes (Fig. 5E), indicating that C3a-peptide indeed enhanced the interactions between MYO10 and C3aR. Co-IP was carried out with C3aR antibody to verify this interaction, and IgG was used as a negative control (Fig. 5G). The results indicated that more MYO10 was immunoprecipitated in C3a-peptide-treated cells compared with untreated cells. Combining all these data, we can conclude that C3a-peptide delivery prompted F-actin aggregation and spindle migration by enhancing the interaction between MYO10 and C3a-peptide/C3aR.

## Discussion

In a clinical ovulation induction cycle, immature human oocytes retrieved from ovaries are considered to have poor potential to develop into offspring and are typically discarded. Effective utilization of immature oocytes can contribute to the cumulative pregnancy rate. Improving IVM of oocytes is essential to enhance the overall in vitro embryo production rates. Understanding oocyte developmental competence remains a key challenge for reproductive biology. As a very important microenvironment for the development of oocytes, FF can provide nutrients and signal stimulus regulation of the folliculogenesis process as well as oocyte growth and development [10]. FF contains growth factors, electrolytes, hormones, amino acids, and unknown factors, with selective filtration of the blood enriched by secretions from the few cells present in the follicle. The in-depth analysis of FF components will help us understand the microenvironment of oocyte development and provide clues for optimizing the oocyte IVM system.

The low-molecular-weight subset of the proteome is termed as the “peptidome,” which includes peptides and small proteins with molecular weights generally less than 10,000 Da. Bioactive peptides thought to be shed from cells to the microenvironment are constantly generated in vivo by active synthesis and by proteolytic processing of larger precursor proteins and often mediate a variety of physiological functions [11]. In this study, we carried out a combined ultrafiltration and LC/MS/MS [12] based peptidomic analysis in order to explore the differentially expressed peptides between mature and immature follicles FF. Interestingly, we screened the differentially expressed peptide C3a from many candidates. The C3a sequence matched that of the complement component C3a, a factor normally linked with the complement and immune systems. In fact, it is no surprise that complement factor B and factors I, C3, and C4 have been found in the FF by proteomics analysis. Next, we investigated the theoretical basis of the biological activity of the C3a-peptide. The C3a-peptide is composed of 25 amino acids that align to residues 672–697 of anaphylatoxin polypeptide (C3a), the third component of complement (C3) [13]. This fragment belongs to the “ANATO” region, corresponding to the anaphylatoxin homologous domain generated enzymatically in serum during activation of complement molecule C3. From an evolutionary perspective, the residues are highly conserved due to natural selection for their function. Usually, the more conserved amino acids in similar proteins from different species are ones that play an essential role in structure and function, and the less conserved ones are in sites that can vary without having a significant effect on function. C3a-peptide and C3a are highly conserved molecules among vertebrates. We therefore believe that C3a-peptide is the active ingredient in FF that promotes the meiotic maturation of oocytes. Currently, traditional perception and functions of complement are under challenge. Accumulating evidence shows that C3 is critical in tissue turnover, the development of bone and cartilage, liver regeneration, and homing of hematopoietic stem cells and neural progenitor cells [14]. Furthermore, recent studies have demonstrated that C3 is important in limb and eye tissue regeneration after injury [15], while C3 and the complement receptor type 3 contribute to synaptic pruning in development and disease [16]. Complement even exerts psychopharmacological control over eating and drinking behavior based on studies in rodents [17]. However, to the best of our knowledge, the roles of C3a in regulating oocyte maturation and meiosis are largely unknown. It is widely accepted that C3a mediates its downstream signaling effects by binding to the C3a receptor (C3aR). C3aR possesses a second extracellular loop of nearly 175 amino acids compared to about 30 amino acids for most G protein-coupled receptors (GPCRs). A two-site C3a-C3aR interaction model is similar to that established for C5a/C5aR. We explored whether C3a-peptide also exerted its effect via C3aR. The following observations helped us to corroborate this theory. C3a-peptide penetrated the membrane and distributed in the cytoplasm of oocytes; C3aR, a seven-transmembrane GPCR, did not locate in the membrane but in the cytoplasm of oocytes at the GV and GVBD stages and co-localized together with β-tubulin on the spindles at the MI and MII stages. Similar results were also observed in 293 T cells (somatic cell line). C3aR antagonist attenuated the increased percentage of oocytes at the MII stage due to overexpression of C3a-peptide. Together with previous observations, these data indeed demonstrated that C3a-peptide promoted IVM of human both mouse oocytes mediated by its receptor, C3aR. Although previous studies reported an unexpected role of complement proteins in early vertebrate development, as shown in one study in which the complement fragment C3a and its receptor C3aR together attracted neural crest cells [18]. These phenomena we observed in this study have not been reported to date.

These findings provide further impetus to pursue the precise molecular details underlying C3aR function. It has been extensively documented that oocyte meiotic maturation undergoes two consecutive asymmetric divisions, and this asymmetry is a consequence of the migration of the microtubule spindle from the cell center toward the closest cortex region [19]. In addition, cytoskeletal remodeling is an important factor during oocyte maturation [19]. We therefore measured various parameters of the oocyte. Summarizing our findings: ❶ C3a-peptide administration promoted the accumulation of F-actin at subcortical regions and in the area surrounding the spindle of the oocyte through C3aR, as evidenced by the intensities of the F-actin clouds around the spindle and located in the subcortex that were significantly diminished once C3aR was inhibited; ❷ the formation of spindles was not impacted by adding C3a-peptide or inhibiting C3aR with morpholinos; ❸C3aR was found to be a crucial molecule responsible for spindle migration due to the fact that C3aR inhibition suppressed spindle migration toward the membrane during oocyte maturation; and ❹ C3a-peptide treatment shortened the mean distance from the spindle to the cortex of the oocyte, and this effect was counteracted by knocking down C3aR, suggesting again that C3a-peptide acted via C3aR. We can therefore conclude that the interaction between C3a-peptide and C3aR was closely associated with F-actin recruitment and the migration of the spindle during meiosis of oocytes. These data reveal that C3a-peptide and C3aR promoted the migration of the MI spindle from the cell center to a sub-cortical location with asymmetric meiotic cell division. Therefore, we believed that the interaction between C3a-peptide and C3aR promoted the growth and development of human and mouse oocytes. Our findings were also supported by previous reports. For example, actin takes on various essential functions during oocyte meiosis [20]; the migration of the spindle toward the oocyte cortex is not dependent on microtubules, but rather on actin filaments [21]; and migration relies only on actin and on a soft cortex for positioning the spindle off-center, and spindle positioning does not depend on microtubules [22].

**Fig. 6:**
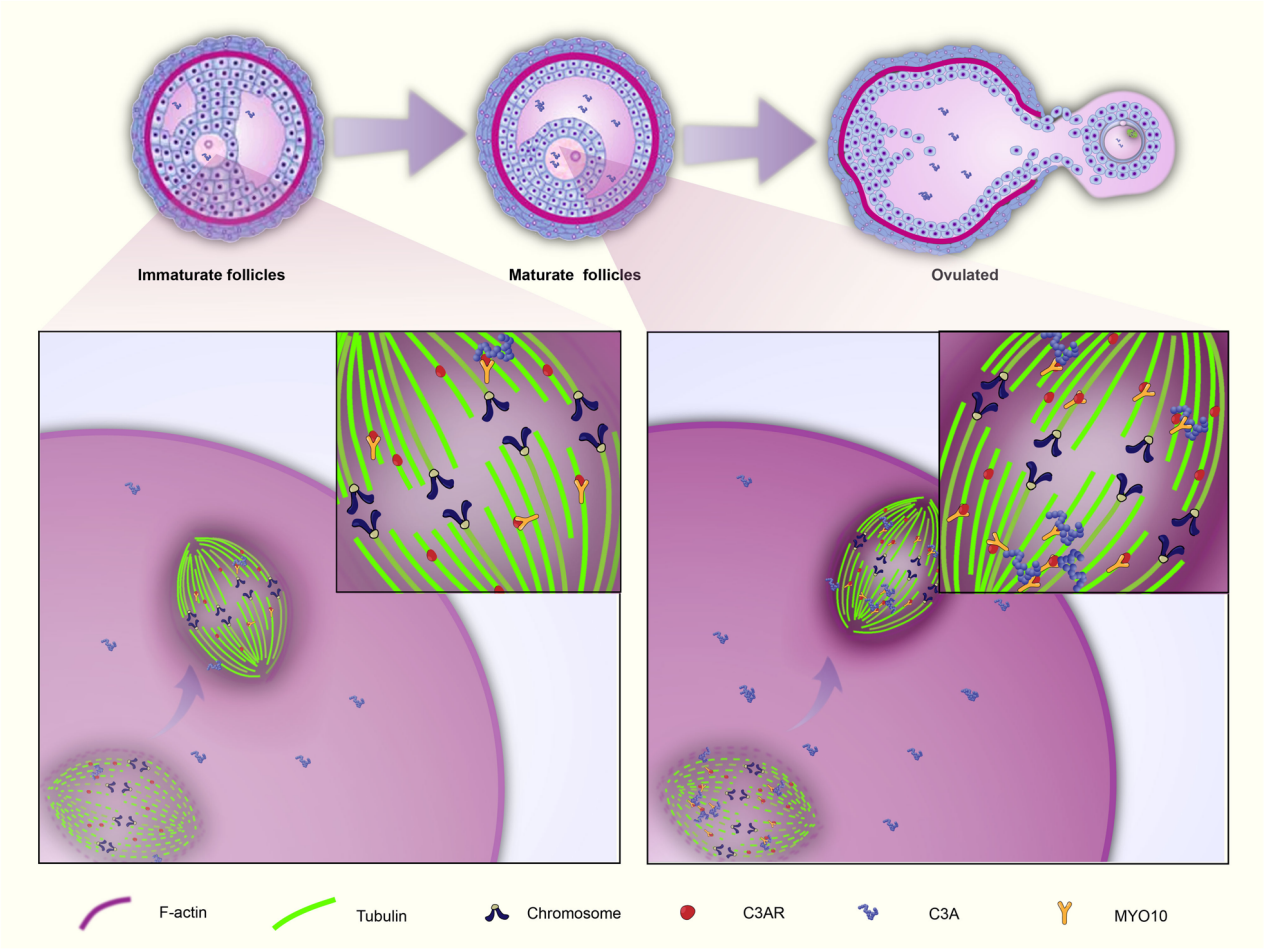
Sketches illustrating the molecular mechanisms of C3a-peptide and C3aR action during meiotic division of oocytes. C3a-peptide accumulates in the follicular fluid during the follicular maturation process and then enters the cytoplasm of the oocytes, where it exerts its effects on maturation by binding to C3aR. C3aR and β-tubulin co-localize on the spindles and directly interact with an intermediate molecule, MYO10. MYO10 contains two domains, the head motor and MyTH4-FERM domain, which bind to F-actin and microtubules, respectively. Our result revealed that C3aR can bind to MYO10. Thus, these molecules collaborate to participate in F-actin aggregation and spindle dynamics in meiotic oocytes

It is also worth considering how C3aR influenced F-actin. Because C3aR and F-actin did not co-localize, we speculated that there was an intermediate molecule linking C3aR and F-actin. MYO10 is a myosin tail homology 4 (MyTH4)-band 4.1, ezrin, radaxin, moesin (FERM) myosin [23], capable of binding to both F-actin via its head motor domain [24] and to microtubules via its MyTH4-FERM domain [25]. Numerous studies have highlighted MYO10 involvement in spindle dynamics, orientation, and positioning in mitotic cells [26]. Importantly, MYO10 was shown to directly link F-actin to spindle microtubules in mouse oocytes and thus participate in spindle off-centering, and MYO10 was found to be indispensable for actin organization, spindle morphogenesis, and positioning in oocytes [27]. In this study, we verified that there was a direct interaction between C3aR as well as MYO10 by using PLA and Co-IP and that this interaction was further boosted by C3a-peptide. Our results should establish a scenario (Fig. 6) describing how C3a-peptide functions in oocyte maturation. C3a-peptide entered the cytoplasm of oocytes and functioned through C3aR. Importantly, C3a-peptide promoted C3aR to directly bind its partner, MYO10, and both of them drove F-actin positioning by surrounding the spindle and centering it to a subcortical region of oocytes in order to assist the migration of the microtubule spindle from the cell center toward the closest cortex. Eventually, meiosis of oocytes could be completed.

A karyotype is the number and appearance of chromosomes. Notably, aneuploidy is the leading cause of congenital birth defects [28] and pregnancy loss [29] and is the main cause of poor pregnancy outcome in IVF. In this study, we assessed and compared the karyotypes between oocytes with or without C3a-peptide treatment. We found that C3a-peptide promoted oocyte maturation in vitro without statistically significant changes in the chromosome aneuploidy rate, either in mouse oocytes or in human oocytes, suggesting that C3a-peptide has the potential to be applied in improving IVM medium in the future.

Finally, it is worth emphasizing that currently many infertile women undergo ART, and the quality of oocytes plays a pivotal role in determining ART outcomes. To further verify the effect of C3a-peptide on oocyte maturation, we specifically selected poorer quality oocytes from mice to evaluate the effect of C3a-peptide. Our results confirmed that C3a significantly improved the percentage of immature oocytes reaching the MII phase, strongly suggesting that further studies should be performed in order to confirm these positive outcomes.

### Perspectives

Although the addition of C3a-peptide promoted the maturation rate of oocytes, many interesting problems need to be explored. For example, we do not know whether C3a-peptide interfered with a self-protection mechanism, such as premature or excessive F-actin polymerization during oocyte development and maturation. We also did not test the fertilization rate and pregnancy rate. We hope that further studies will be performed in order to enable C3a-peptide application in clinical treatment.

## Conclusions

Our results provide a new perspective for improving IVM culture systems by applying FF components and also provide molecular insights into the physiological function of C3a-peptide.

## Methods

### Ethical statement

This study was approved by the Commission for Medical Ethics of the Affiliated Obstetrics and Gynecology Hospital of Nanjing Medical University, China (code ML6214). Patients were fully informed, and consent was obtained before FF retrieval. With the approvement of the Committee of Medical Ethics (2021H041), immature human oocytes were collected during ART cycles in Reproductive Medicine Center of the First Affiliated Hospital of Anhui Medical University. The Animal Care and Use Committee of The Nanjing medical university reviewed and approved all of the procedures used in this study (2015–249). Informed consent was obtained from all patients participating in this study. All experiments were performed according to good practices of laboratory animal management.

### Study population

The FFs derived from five pairs of women who underwent an IVF procedure due to male factor infertility were analyzed in this study. In addition, a total of 320 immature oocytes derived from other 103 women were used to verify the effect of CO3-pepitide on oocyte maturation. Basically, all patients underwent controlled ovarian hyperstimulation as part of IVF treatment. Women participating in this study underwent a gonadotropin-releasing hormone antagonist (GnRH-ant) protocol. On day 2 or day 3 of the menstrual cycle, the initial dose of rFSH was administered, ranging from 112.5 to 200 IU per day. A GnRH-ant (cetrorelix acetate, Baxter Oncology GmbH, Germany) at a daily dose of 250 µg was begun after 4–5 days following rFSH initiation until the day of hCG administration. The initial dose of rFSH was based on age, antral follicle count, and basal FSH levels, and the ovarian response was monitored through serum sex steroids and serial transvaginal ultrasound examinations during stimulation. Gonadotropin doses were adjusted when needed. Human menopausal gonadotropin (Lizhu, China) was used.

### Follicular fluid (FF) sample collection

At 34–36 h after intramuscular injection of hCG, oocytes and their corresponding FF were retrieved using transvaginal ultrasound-guided aspiration. Ultrasonography was used to evaluate follicular development from day 5 of stimulation until the day of follicular maturation. Follicle size was determined based on the average follicular diameter. Two FF samples from each patient were obtained, including mature follicles (17–22 mm) and matched-immature follicles (8–13 mm), resulting in 10 samples in total. These samples were further referred to using the following abbreviations: P1 − P5, patient number 1 − 5; im and m referred to immature FF and mature FF, respectively. Samples were stored separately. The FF samples were centrifuged at 12,000 g for 10 min to remove blood, and the supernatant was stored in one additional tube and frozen at − 80 °C for subsequent studies.

### Peptide extraction and data analysis

The peptide extraction and identification were performed by BGI-Shenzhen Company. First, the proteins in the FF were diluted with 8 M urea to 10 mg/mL in 0.1 M Tris–HCl, pH 8.5. After cooling at room temperature, the proteins were alkylated with 55 mM iodoacetamide in the dark at room temperature for 45 min [30]. The concentrations of FF samples were determined using Bradford reagent (Sigma, St. Louis, MO, USA) as a standard. Sodium dodecyl-sulfate polyacrylamide gel electrophoresis was carried out according to Laemmli [31]. To focus on peptides, only the fractions with mass weights less than 10 kDa were analyzed to uncover the complexity of FF. Then, the samples were fractionated in a Strata-X C18 column (Thermo Scientific) previously conditioned with methanol. The eluate was dried in a SCANVAC (Denmark) concentrator. The dried eluates were stored at − 20 °C for further nano-LC–MS/MS analysis.

### LC–MS/MS analysis and peptide identification

LC–MS/MS was performed on a Prominence nano-HPLC system (Shimadzu, Tokyo, Japan) coupled with a Q-Exactive (Thermo Fisher Scientific, Waltham, MA, USA). The peptides were separated by nano-LC on an in-house packed Ultimate XB-C18 column (Welch Materials, Ellicott, MD, USA) at a flow rate of 300 nL/min. Each fraction was dissolved in 0.1% formic acid and then injected and eluted using a gradient of 5–30% solvent B (95% acetonitrile, 0.1% formic acid) over 40 min. The mass spectrometers were operated in a data-dependent mode, automatically switching between MS and MS2 acquisition. Survey full scan MS spectra (m/z 350–1800) were acquired in the Orbitrap with a resolution of 70,000. The 20 most intense ions were sequentially isolated and fragmented by high energy collisional dissociation. Peptides with unassigned charge states as well as with charge states less than + 2 or more than + 6 were excluded from fragmentation. Fragment spectra were recorded in the Orbitrap mass analyzer with a resolution of 17,500. Dynamic exclusion was enabled with a repeat count of two and exclusion duration of 8 s.

### Construction of phylogenetic trees

Sequences for C3a-peptide and C3a were retrieved from the UniProt Knowledgebase. Sequence alignments were carried out using MUSCLE from the MEGA5 package. The maximum likelihood method was used to reconstruct the phylogenetic trees. The best-fit substitution model, tree reconstruction, visualization, and annotation were chosen using MEGA5. Bootstrap support values were based on 10,000 replicates. Each ancestral amino acid and its associated probability value at every site of each node were retrieved from an Excel file supplied by MEGA5 and used to reconstruct the most probable ancestral sequence for each node of the tree.

### Mouse oocyte collection and culture

A total of 300 ICR mice used in this study were housed under specific pathogen-free environmental conditions with free access to water and food, a temperature of 20–22 °C, 50–70% humidity, and a 12-h light/dark cycle at the Animal Core Facility of Nanjing Medical University. The animal care and experimental procedures were conducted according to the guidelines of the Animal Research Committee of Nanjing Medical University. For the collection of oocytes, 3–6-week-old ICR mice were killed by cervical dislocation to collect ovaries in M2 medium. GV intact oocytes were picked by a pipetted tube under a stereoscope. Groups of 25–30 oocytes were placed in 30-μL droplets of the M16 culture medium under mineral oil in a cell culture dish and maintained at 37 °C in a 5% humidified CO_2_ atmosphere for maturation in vitro.

### Peptide treatment

Seven peptides were synthetized by Genscript Company and dissolved in M16 medium as stock solutions. The stock solutions were further diluted in the M16 medium as working solutions. To test the toxic effects of exposure to each peptide on the meiotic maturation of oocytes, we evaluated the extrusion rate of GVBD and the first polar body using three peptide concentrations. To confirm the promotive effect of the peptides, oocytes were randomly assigned to four groups: the untreated group and three experimental groups treated with one of the three peptide concentrations that was established according to the maximum solubility of each peptide.

### Morpholino experiments

The antisense morpholino oligonucleotide spanning C3aR gene (5′- TGGTGTCAGCATCGAAGACTCCAT-3′) and a missense N-morpholino control oligonucleotide (5′- CCTCTTACCTCAGTTACAATTTATA-3′) (Gene Tools, LLC) was diluted by water (Sigma Chemical Co., St. Louis, MO) to give a concentration of 2 mM. GV oocytes were microinjected with 5–10 pl MO (Gene Tools, LLC) in M2 medium containing 2.5 μM Milrinone (Sigma) for preventing oocyte GV break down. Oocytes were incubated in M2 medium containing 2.5 μM Milrinone for 20 h, washed 5 times in fresh M2 medium, and then cultured in fresh M2 medium to resume meiosis. The oocytes cultured for 7 h and 14 h (for MI and MII stage respec tively) were collected for subsequent experiments.

### In Vitro* Maturation (IVM) of Human Oocytes*

Immature human oocytes in intracytoplasmic sperm injection cycles were collected in this study to investigate the role of the C3a-peptide in the IVM process. As previously described, the basic IVM medium was composed of tissue culture medium 199 supplemented with 0.22 mM pyruvic acid, 0.075 IU/mL FSH, 0.5 IU/mL HCG, 0.1 mg/mL 17β-estradiol, 0.6 g/L penicillin, 0.6 g/L streptomycin, and 20% (v/v) of the patient’s inactivated serum. The C3a- peptide-supplemented IVM medium was prepared by adding 1.5 μM C3a-peptide to the general IVM medium. We designed and conducted the self-controlled study by randomly assigning the GV oocytes from the same patients (more than two immature oocytes) into the basic IVM or C3a-peptide supplemented IVM medium.

### Immunofluorescence and confocal microscopy

At room temperature, the oocytes were fixed with 4% paraformaldehyde for 30 min. After washing, the oocytes were permeabilized in Dulbecco's phosphate buffered saline (PBS) supplemented with 0.5% Triton X‐100 for 20 min and blocked in PBS containing 1% bovine serum albumin for 1 h. Then, the oocytes were incubated with different primary antibodies at 4 °C overnight. Primary antibodies included phalloidin (A12379, ThermoFisher), anti-α-tubulin antibody (GTX76511, GeneTex), and C3aR antibody (Sc-133172, Santa Cruz Biotechnology, Inc; or Abnova, H00010454‐PW1). The specificity of C3aR primary antibody was confirmed through peptide blocking assays. In brief, the blocking peptide is synthetized (Genscript Biotechnology, China). The C3aR antibody alone as well as the mixture of blocking peptide and the primary antibody (the final concentration of blocking peptide is 20 ng/ul) were added to their respective oocytes for parallel staining experiments. The oocytes were then incubated with an appropriate secondary antibody at room temperature for 1 h. The DNA was counterstained with Hoechst 33,342 (DAPI, C1022; Beyotime Institute of Biotechnology, Shanghai, China) for 20 min. After washing three times, the oocytes were placed on a glass slide in a 5-μL drop of antifade mounting medium. Representative images were captured with a laser-scanning confocal microscope (Zeiss LSM 800 Confocal Laser Scanning Microscope).

### Oocyte karyotyping

Oocytes were exposed to Tyrode’s buffer (pH 2.5) for 30 s at 37 °C to remove the zona pellucida. After recovery in M2 medium (Millipore Sigma) for 10 min, the oocytes were fixed in a drop of 1% paraformaldehyde and 0.15% Triton X-100 on a glass slide. After air drying, the oocytes were incubated with CREST antibody (Antibodies Incorporated; 1:500, Cat#: 15–234) overnight at 4 °C and then incubated with a secondary antibody for 1 h for kinetochore labeling. Chromosomes were stained with DAPI, and the samples were examined under a laser scanning confocal microscope.

### Plasmids, cell transfection, and protein expression

We amplified human C3a-peptide and C3a protein by cloning them into a pcDNA3.1 vector with a 3XFlag tag. 293 T cells were grown in Dulbecco's modified Eagle's medium supplemented with 10% fetal bovine serum and maintained at 37 °C in a humidified incubator with 5% CO_2_. Upon nearing confluence, the cells were dissociated enzymatically with trypsin–EDTA and passaged. The 293 T cells were transfected with plasmids expressing the C3a peptide or C3a protein using Lipofectamine 3000 (Invitrogen). The cells were lysed in lysis buffer containing protease inhibitors and subjected to Western blotting with antibodies against Flag.

The pcDNA3.1-3xmScarletI_Utrch plasmid was constructed by GENERAL BIOL. The probe was linearized and transcribed into mRNA in vitro, and mRNA was microinjected into the oocytes at the GV stage. Then, the oocytes were cultured in a medium supplemented with or without C3a-peptide. The distribution of F-actin in the oocytes at the GVBD and MII stages was observed under a confocal microscope after incubating the oocytes for 3 h and 14 h, respectively.

### Immunoprecipitation

Immunoprecipitation experiments were performed using a Pierce Crosslink immunoprecipitation kit (Pierce, #26,147) according to the instructions of the manufacturer. Briefly, mouse oocytes were collected and lysed in buffer (0.025 M Tris, 0.15 M NaCl, 0.001 M EDTA, 1% NP‐40, 5% glycerol; pH 7.4), and the lysates were cleared by centrifugation (10,000 g for 10 min) at 4 °C. The supernatant was transferred into a new tube, and anti-Flag antibody cross-linked with protein A/G plus agarose beads was added to the samples and incubated at 4 °C overnight. After elution, the samples were analyzed with Western blotting. C3aR IP-WB antibody pair (Abnova, H00010454-PW1) was used for immunoprecipitation.

### Proximity Ligation Assay (PLA)

To detect the protein–protein interactions in situ, a DuoLink in situ PLA kit (Merck) was used according to the manufacturer's protocol. After fixation and permeabilization, oocytes were incubated in blocking solution and immunolabeled (overnight, 4 °C) with primary antibodies diluted in the DuoLink® antibody diluent: rabbit anti-C3AR and anti-MYO10 (ab58699, Abcam); negative controls excluded one primary antibody or both. Then, the secondary antibodies with attached PLA probes were used. Finally, the DNA was stained with DAPI. Protein complex signals were observed using a Carl Zeiss LSM 800 confocal microscope with ZEN navigation software.

### Statistical analysis

All experiments were repeated at least three times for statistical analysis, and data from one representative experiment are shown, unless otherwise stated. Quantifications were presented as mean values ± standard deviations of mean (SD). Statistical analyses were performed with independent sample two-sided T-test or the non-parametric Mann Whitney U Test for comparing two samples. For a comparison of more than two group means, single factor ANOVA analysis (LSD or S–N-K post hoc multiple analysis, Tamhane and Games Howell or non-parametric test) were used according to normal distribution or not. Statistical significance was determined as indicated in the figure legends. *P* values < 0.05 were regarded as significant (*P < 0.05; **P < 0.01; ***P < 0.001). Sample sizes were chosen according to studies using similar methods and were comparable with what is routinely employed in this field. More details of experimental statistics (such as sample size (n), description of sample collection, and the number of replicates) are presented in the corresponding Results section and corresponding figure legends. There was no statistical method used to predetermine the sample size.

### Supplementary Information

**Additional file 1.** The raw mass spectrometry data. Short peptides analysis.

**Additional file 2:**  **Fig. S1.** Screening effective short peptides for oocyte maturation. According to the percentages at the GV, GVBD, MI, and MII stages.

**Additional file 3: Fig. S2.** C3a-peptides are conserved between mice and humans. C3a-peptides are conserved between mice and humans.

**Additional file 4: Fig. S3.** C3aR is expressed in oocytes, and C3aR and β-tubulin are entirely co-localized on the spindles. Immunofluorescence image shows location and co-location between C3aR andβ-tubulin.

**Additional file 5.** Original data. Original membranes. Original results of WB.

**Additional file 6.** Numbers of samples and numbers of experimental repetitions. Oocyte culture results. Calculating the percentages at the GV, GVBD, MI, and MII stages.

## Data Availability

All data generated or analysed during this study are included in this published article and its supplementary information files. The analyzed datasets and samples have been shown in Additional files 1, 2, 3, 4, 5 and 6.

## Abbreviations

IVM: In vitro maturation
FF: Follicular fluid
GV: Germinal vesicle
GVBD: Germinal vesicle breakdown
IVF: In vitro fertilization
IVM: In vitro maturation
FSH: Follicle stimulating hormone
LC–MS: Liquid chromatography-mass spectrometry
PBS: Phosphate buffered saline
GRAVY: Grand average of hydropathicity
PI: Isoelectric point
APPOA1: Apolipoprotein A-I isoform 1
C3a: Complement C3 chain A
IGHG2: Immunoglobulin heavy constant gamma 2
FETUA: Alpha-2-HS-glycoprotein
Co-IP: Co-immunoprecipitation
PLA: Proximity ligation assay

## References

[1] Yang Q, Zhu L, Wang M, Huang B, Li Z, Hu J (2021). Analysis of maturation dynamics and developmental competence of in vitro matured oocytes under time-lapse monitoring. Reprod Biol Endocrinol.

[2] Trebichalska Z, Kyjovska D, Kloudova S, Otevrel P, Hampl A, Holubcova Z (2021). Cytoplasmic maturation in human oocytes: an ultrastructural study dagger. Biol Reprod.

[3] Jie H, Zhao M, Alqawasmeh OAM, Chan CPS, Lee TL, Li T, et al. In vitro rescue immature oocytes - a literature review. Hum Fertil. 2022;25(4):640–50. 10.1080/14647273.2021.1876932 33508986

[4] Dahan MH, Tan SL, Chung J, Son WY (2016). Clinical definition paper on in vitro maturation of human oocytes. Hum Reprod.

[5] McGee EA, Hsueh AJ (2000). Initial and cyclic recruitment of ovarian follicles. Endocr Rev.

[6] Kumariya S, Ubba V, Jha RK, Gayen JR (2021). Autophagy in ovary and polycystic ovary syndrome: role, dispute and future perspective. Autophagy.

[7] Dumesic DA, Meldrum DR, Katz-Jaffe MG, Krisher RL, Schoolcraft WB (2015). Oocyte environment: follicular fluid and cumulus cells are critical for oocyte health. Fertil Steril.

[8] Lazzarino G, Pallisco R, Bilotta G, Listorti I, Mangione R, Saab MW, et al. Altered Follicular Fluid Metabolic Pattern Correlates with Female Infertility and Outcome Measures of In Vitro Fertilization. Int J Mol Sci. 2021;22(16):8735. 10.3390/ijms22168735 PMC8395780 34445441

[9] Soderberg O, Gullberg M, Jarvius M, Ridderstrale K, Leuchowius KJ, Jarvius J (2006). Direct observation of individual endogenous protein complexes in situ by proximity ligation. Nat Methods.

[10] Park MR, Gupta MK, Lee HR, Das ZC, Uhm SJ, Lee HT (2011). Possible involvement of Class III phosphatidylinositol-3-kinase in meiotic progression of porcine oocytes beyond germinal vesicle stage. Theriogenology.

[11] Yadikar H, Johnson C, Pafundi N, Mouhawasse E, Nguyen L, Torres I, et al. Novel Peptidomic Approach for Identification of Low and High Molecular Weight Tauopathy Peptides Following Calpain Digestion, and Primary Culture Neurotoxic Challenges. Int J Mol Sci. 2019;20(20):5213. 10.3390/ijms20205213 PMC6829287 31640160

[12] Tirumalai RS, Chan KC, Prieto DA, Issaq HJ, Conrads TP, Veenstra TD (2003). Characterization of the low molecular weight human serum proteome. Mol Cell Proteomics.

[13] Markiewski MM, Lambris JD (2007). The role of complement in inflammatory diseases from behind the scenes into the spotlight. Am J Pathol.

[14] Klos A, Wende E, Wareham KJ, Monk PN. International Union of Basic and Clinical Pharmacology. [corrected]. LXXXVII. Complement peptide C5a, C4a, and C3a receptors. Pharmacol Rev. 2013;65(1):500–43. 10.1124/pr.111.005223 23383423

[15] Kimura Y, Madhavan M, Call MK, Santiago W, Tsonis PA, Lambris JD (2003). Expression of complement 3 and complement 5 in newt limb and lens regeneration. J Immunol.

[16] Stephan AH, Barres BA, Stevens B (2012). The complement system: an unexpected role in synaptic pruning during development and disease. Annu Rev Neurosci.

[17] Ohinata K, Takagi K, Biyajima K, Kaneko K, Miyamoto C, Asakawa A (2009). Complement C5a stimulates food intake via a prostaglandin D(2)- and neuropeptide Y-dependent mechanism in mice. Prostaglandins Other Lipid Mediat.

[18] Carmona-Fontaine C, Theveneau E, Tzekou A, Tada M, Woods M, Page KM (2011). Complement fragment C3a controls mutual cell attraction during collective cell migration. Dev Cell.

[19] Duan X, Sun SC (2019). Actin cytoskeleton dynamics in mammalian oocyte meiosis. Biol Reprod.

[20] Uraji J, Scheffler K, Schuh M. Functions of actin in mouse oocytes at a glance. J Cell Sci. 2018;131(22):jcs218099. 10.1242/jcs.218099 30467138

[21] Yi K, Rubinstein B, Unruh JR, Guo F, Slaughter BD, Li R (2013). Sequential actin-based pushing forces drive meiosis I chromosome migration and symmetry breaking in oocytes. J Cell Biol.

[22] Longo FJ, Chen DY (1985). Development of cortical polarity in mouse eggs: involvement of the meiotic apparatus. Dev Biol.

[23] Hirano Y, Hatano T, Takahashi A, Toriyama M, Inagaki N, Hakoshima T (2011). Structural basis of cargo recognition by the myosin-X MyTH4-FERM domain. EMBO J.

[24] Homma K, Saito J, Ikebe R, Ikebe M (2001). Motor function and regulation of myosin X. J Biol Chem.

[25] Weber KL, Sokac AM, Berg JS, Cheney RE, Bement WM (2004). A microtubule-binding myosin required for nuclear anchoring and spindle assembly. Nature.

[26] Sandquist JC, Larson ME, Woolner S, Ding Z, Bement WM (2018). An interaction between myosin-10 and the cell cycle regulator Wee1 links spindle dynamics to mitotic progression in epithelia. J Cell Biol.

[27] Crozet F, Da Silva C, Verlhac MH, Terret ME. Myosin-X is dispensable for spindle morphogenesis and positioning in the mouse oocyte. Development. 2021;148(7):dev199364. 10.1242/dev.199364 PMC8077531 33722900

[28] Hassold T, Hunt P (2001). To err (meiotically) is human: the genesis of human aneuploidy. Nat Rev Genet.

[29] Bauld R, Sutherland GR, Bain AD (1974). Chromosome studies in investigation of stillbirths and neonatal deaths. Arch Dis Child.

[30] Duan Z, Cao R, Jiang L, Liang S (2013). A combined de novo protein sequencing and cDNA library approach to the venomic analysis of Chinese spider Araneus ventricosus. J Proteomics.

[31] Laemmli UK (1970). Cleavage of structural proteins during the assembly of the head of bacteriophage T4. Nature.

