# Supplementary material for: Follicular fluid C3a-peptide promotes oocyte maturation through F-actin aggregation
Source: BMC Biol. 2023 Dec 8;21:285. doi: 10.1186/s12915-023-01760-6 (PMC10709936; doi:10.1186/s12915-023-01760-6)
Supplement: Supplementary file 2 — Additional file 2: Fig. S1. Screening effective short peptides for oocyte maturation. According to the percentages at the GV, GVBD, MI, and MII stages. [file 12915_2023_1760_MOESM2_ESM.pdf]

Figure 1S. Screening effective short peptides for oocyte maturation according to the percentages at the GV, GVBD, MI, and MII stages.

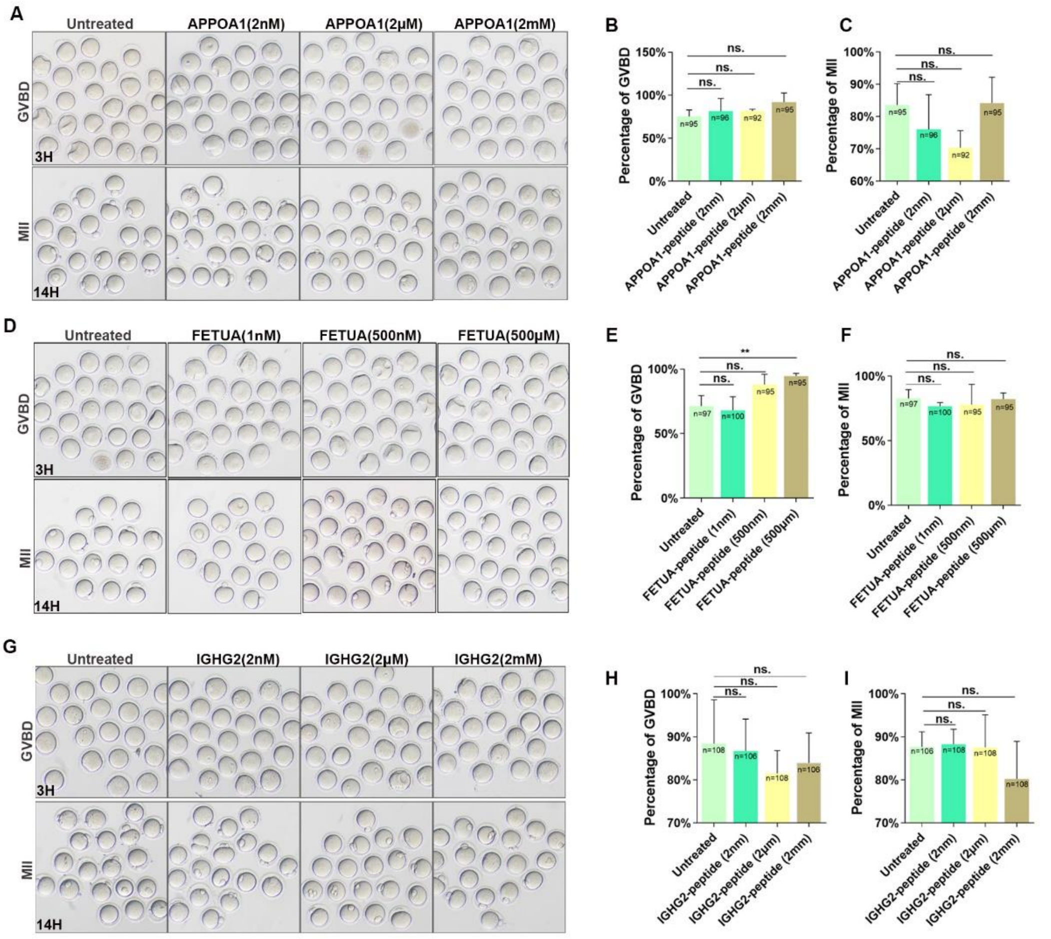

The synthetic short peptides were tested by adding them at various concentrations to the medium. The short peptides were C3a-peptide, APPOA1, FETUA, and IGHG2. In addition to C3a-peptide, three other short peptides were evaluated, but they did not promote the growth and development of mouse ovarian follicles at either the GVBD or MII stages.
