# Supplementary material for: Follicular fluid C3a-peptide promotes oocyte maturation through F-actin aggregation
Source: BMC Biol. 2023 Dec 8;21:285. doi: 10.1186/s12915-023-01760-6 (PMC10709936; doi:10.1186/s12915-023-01760-6)
Supplement: Supplementary file 3 — Additional file 3: Fig. S2. C3a-peptides are conserved between mice and humans. C3a-peptides are conserved between mice and humans. [file 12915_2023_1760_MOESM3_ESM.pdf]

Figure 2S. C3a-peptides are conserved between mice and humans.

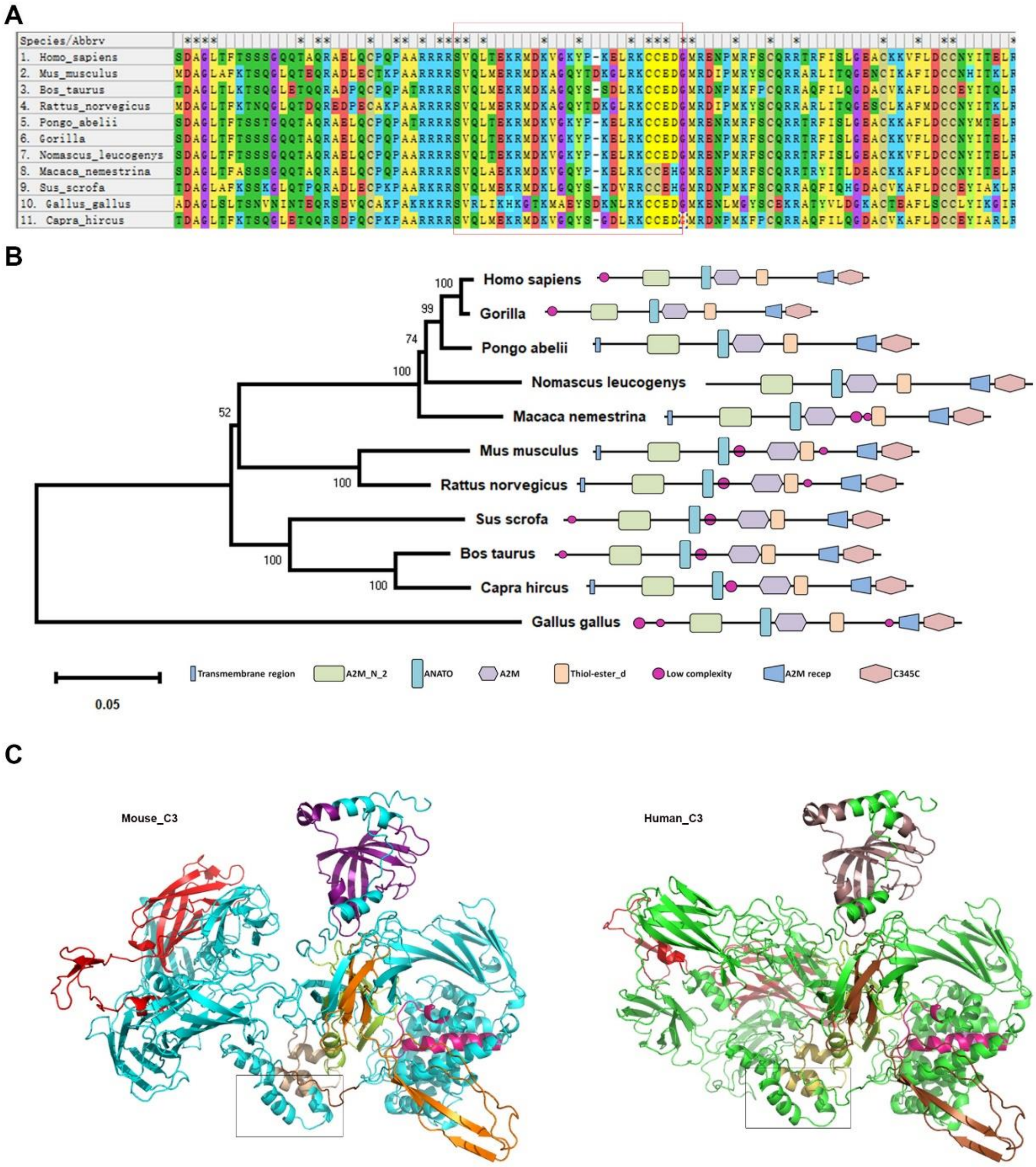

(A) Alignment of the amino acid sequences of eutherian mammalian C3a in 11 species. The sequences of C3a-peptide derived from 11 species are shown in the red box. The computer program MEGA was used to generate this figure.

To elucidate the protein or proteins from which the short peptides were derived, the short peptide sequences were compared with those in the GenBank

database using BLAST. The sequence of the C3a-peptide matched that of human complement C3a. Moreover, the amino acid sequences of complement C3a protein derived from 11 species were analyzed with ClustalX_v2.0 using the default parameters; the output was a graphical and text alignment. The 11 species ranged from birds, such as *Gallus*, to higher mammals, such as gorillas and humans. The amino acids located in a column marked by asterisks are exactly the same in all 11 species. The C3a-peptide derived from the 11 species is cropped in a red box. The results revealed that the C3apeptide was highly homologous between mice and humans. **(B)** Maximum likelihood phylogenetic tree of the 11 eutherian mammalian C3a-peptide/C3a sequences. The MEGA program was employed. We built maximum likelihood trees with MEGA5 [52] from the amino acid composition of complement C3 of 11 species. The resulting phylogenetic tree consists of two major mammalian vertebrate clusters. **(C)** Position of the C3a-peptide in computational 3D structures of human and mouse C3a. The figure was generated using PyMol (The PyMol Molecular Graphic System, Version 1.3, Schrödinger, LLC). The complement C3-related structural domains were found to be conserved in humans and mice. We also compared and analyzed the spatial configurations of complement C3 between mice and humans. The black boxes enclose the C3a-peptides of mice and humans, and their spatial conformation is essentially the same.
