# Supplementary material for: Follicular fluid C3a-peptide promotes oocyte maturation through F-actin aggregation
Source: BMC Biol. 2023 Dec 8;21:285. doi: 10.1186/s12915-023-01760-6 (PMC10709936; doi:10.1186/s12915-023-01760-6)
Supplement: Supplementary file 4 — Additional file 4: Fig. S3. C3aR is expressed in oocytes, and C3aR and β-tubulin are entirely co-localized on the spindles. Immunofluorescence image shows location and co-location between C3aR andβ-tubulin. [file 12915_2023_1760_MOESM4_ESM.pdf]

Figure 3S. C3aR is expressed in oocytes, and C3aR and β-tubulin are entirely co-localized on the spindles

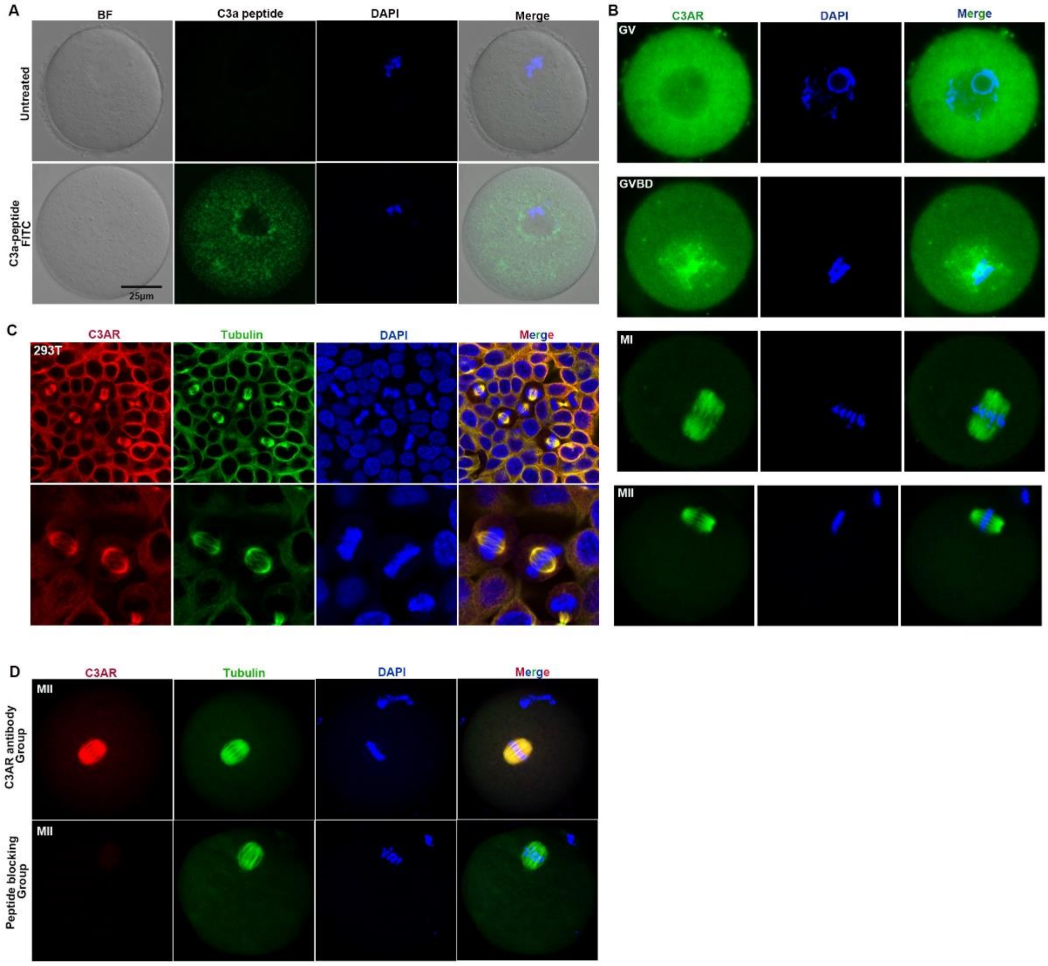

(A) Immunofluorescence image shows that synthetic C3a-peptide labeled by FITC distributed evenly in the cytoplasm of mouse oocytes. **(B)** C3aR expression in the developing oocyte was verified by immunofluorescence staining with a C3aR antibody derived from a different company. **(C)** Double immunofluorescence staining with C3aR and tubulin antibodies verified that the C3aR and β-tubulin positive signals were entirely co-localized on the spindles in 293T cells, a somatic cell line. **(D)** The specificity of primary antibody C3aR is confirmed through peptide blocking assays.
