# Supplementary material for: Follicular fluid C3a-peptide promotes oocyte maturation through F-actin aggregation
Source: BMC Biol. 2023 Dec 8;21:285. doi: 10.1186/s12915-023-01760-6 (PMC10709936; doi:10.1186/s12915-023-01760-6)

1. The Original gel/blots of Figure 3A

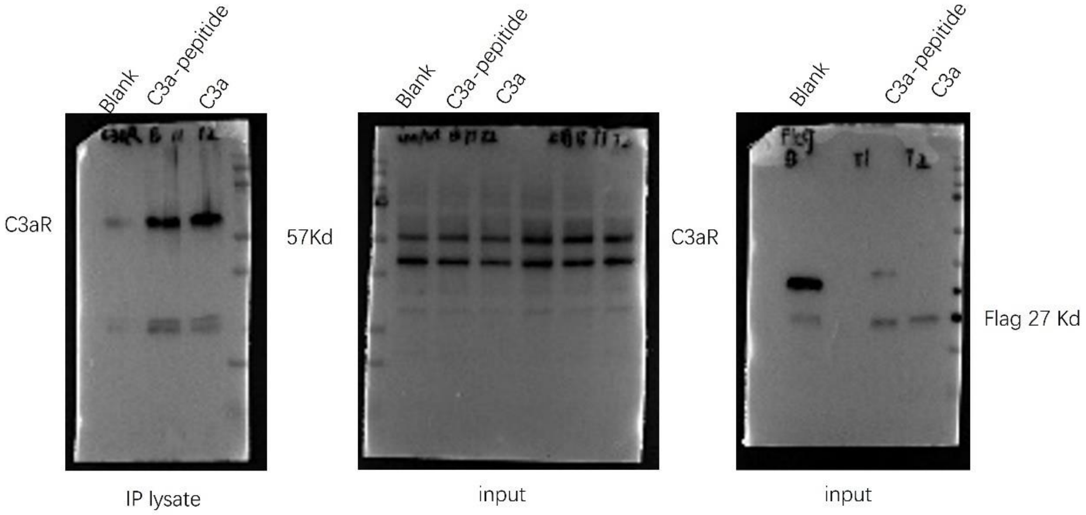

2. The Original gel/blots of Figure 4B

The first time experimental membrane

The second time experimental membrane

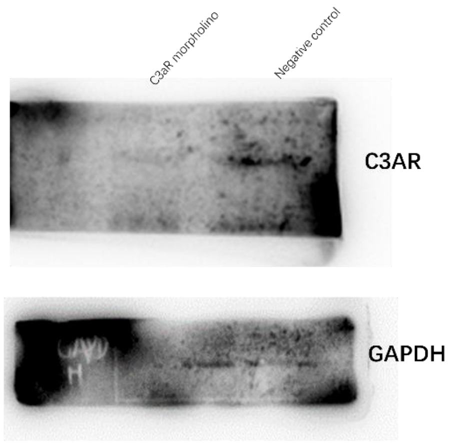

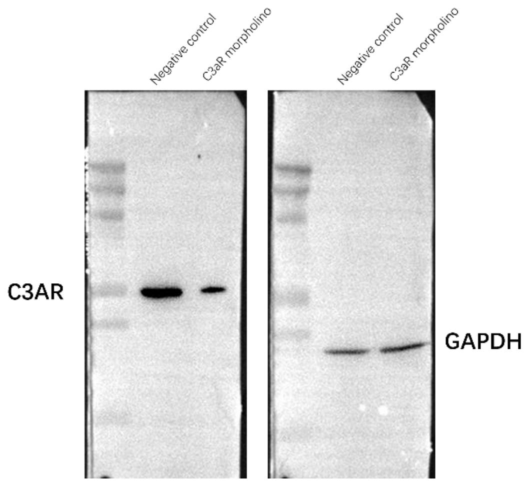

3. The Original gel/blots of Figure 5G

IP ： Myo10

input

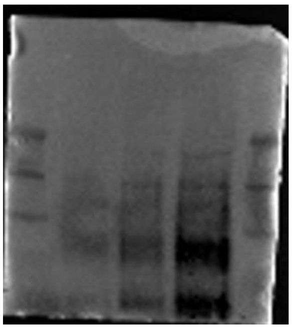
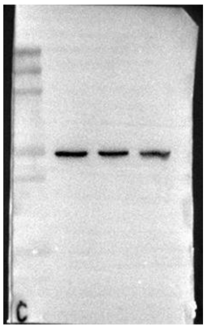

Supplement: Supplementary file 6 — Additional file 6. Numbers of samples and numbers of experimental repetitions. Oocyte culture results. Calculating the percentages at the GV, GVBD, MI, and MII stages. [file 12915_2023_1760_MOESM6_ESM.pdf]
